# Utilizing two-dimensional monolayer and three-dimensional spheroids to enhance radiotherapeutic potential by combining gold nanoparticles and docetaxel

**DOI:** 10.1186/s12645-023-00231-5

**Published:** 2023-10-19

**Authors:** Kyle Bromma, Wayne Beckham, Devika B. Chithrani

**Affiliations:** 1 Department of Physics and Astronomy, University of Victoria, Victoria, BC Canada; 2 British Columbia Cancer, Victoria, BC Canada; 3 Centre for Advanced Materials and Related Technologies (CAMTEC), University of Victoria, Victoria, BC Canada; 4 Division of Medical Sciences, University of Victoria, Victoria, BC Canada

**Keywords:** Gold nanoparticles, Docetaxel, Radiotherapy, Spheroids, Cancer, Nanomedicine

## Abstract

**Background:**

Much in vitro research on the applicability of gold nanoparticles (GNPs) in cancer treatment has been focused on two-dimensional (2D) monolayer models. To improve this, we explored the effect of the combination of GNPs and docetaxel (DTX) with radiotherapy (RT) in a more complex three-dimensional (3D) spheroid that can better mimic a real tumour microenvironment.

**Methods:**

Two cell lines, prostate cancer LNCaP and cervical cancer HeLa, were grown in monolayer and spheroids. Cells were dosed with GNPs at a concentration of 10 $\mathrm{\mu g}/\mathrm{mL}$ and with DTX at a dose that inhibited growth-rate by 50%. Samples were irradiated 24 h after drug dosing with 2 Gy, 5 Gy, or 10 Gy using a 6 MV beam. Monolayer cells had the DNA double-strand breaks (DSBs) probed 24 h post-radiation, and cell proliferation observed over 7 days. Spheroid proliferation was monitored over 14 days along with spheroid volume measurements.

**Results:**

In DTX and GNP-treated monolayer samples, there is decreased survival after irradiation with 5 and 10 Gy of 16–24% and an increase in DSBs of 91.6–109.9%, compared to DTX. In spheroids, GNPs decreased the surviving cells by 10.54–15.61% compared to control, while GNPs and DTX decreased survival by 20.9–31.04%. There is reduced spheroid volume 14 days after treatment with the triple combination.

**Conclusions:**

Combining GNPs and DTX leads to a synergistic radiosensitization effect in spheroids, which can better mimic the tumour microenvironment. Testing treatment modalities with spheroids and RT may allow a quicker translation to the clinic.

**Supplementary Information:**

The online version contains supplementary material available at 10.1186/s12645-023-00231-5.

## Introduction

When optimizing the treatment of a combined therapy platform, a two-dimensional (2D) monolayer of cells has been largely utilized. However, the use of a flat surface of cells has been criticized for being an inaccurate view of the complex, heterogeneous tumor microenvironment (TME) that exists within a malignancy (Pampaloni et al. 2007). There is a loss of tissue-specific architecture, intra-cellular contact, and cell–cell communication that is present in a tumour. Furthermore, a monolayer of cells is grown on a flat substrate that allows the cells to stretch, forming an increased surface area that is directly exposed to culture medium, including drug treatments (Nath and Devi 2016). There is compelling evidence that the TME plays a critical role in cancer development and progression, which monolayer culture can not properly model (Quail and Joyce 2013). These limitations, however, give way to the advantage that a monolayer of cells provides, such as low cost, high throughput, and a high degree of control over environmental factors (Edmondson et al. 2016). Aside from these benefits, there may be many inconsistencies when it comes to comparing monolayer culture to in vivo experimental results, and thus a better in vitro solution should be explored.

**Fig. 1:**
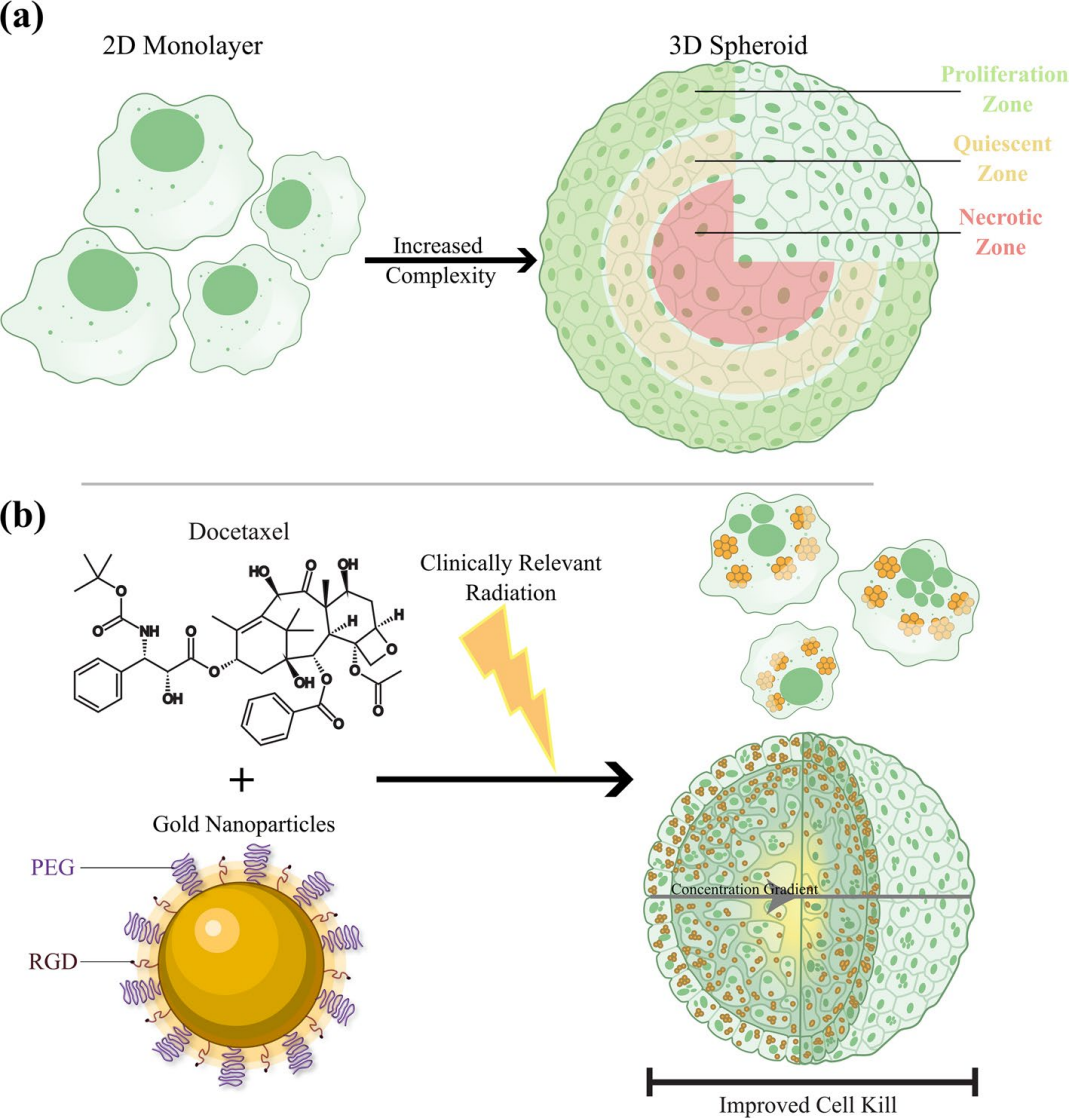
Improved cell kill with combined cancer therapy. **a** Multicellular spheroid is a three-dimensional in vitro tissue structure that can better approximate the complex heterogeneous in vivo tumour microenvironment. This includes modelling the ECM, which has a proliferation zone on the outer edge of the spheroid, a quiescent zone in the middle of the spheroid, where the cancer does not grow effectively, and possible necrotic core, where the cells lack the nutrients to survive due to the concentration gradient and are either hypoxic or are dead. **b** With the addition of GNPs and DTX, the cancer cells become more sensitized to radiation, and we see an improved cell kill response. This occurs even in clinically relevant 6 MV radiation

By growing a three-dimensional (3D) structure in the lab, we can bridge the gap between monolayer and in vivo approaches and improve the physiological relevance of in vitro assays. There are various types of 3D culture models that can be implemented in the lab, such as tumour tissue explants, tumor grown on chips, and multicellular spheroids (Nath and Devi 2016). While various models have benefits, multicellular spheroids are one of the most well-characterized models of in vitro cancer. Spheroids are aggregates of either a single or multiple cell types, which have 3D cell–cell contact and proliferate in a geometry that is very similar to that observed in vivo. Thus, spheroids can stimulate the production of an extracellular matrix (ECM) (Dubessy et al. 2000). There are many benefits that can be ascertained from the 3D structure of spheroids. This includes the establishment of a gradient of nutrients, ${\mathrm{O}}_{2}$, and pH as they grow larger. Furthermore, a cross section of a spheroid reveals concentric rings of a heterogeneous cell population that is commonly observed in vivo, as can be partially observed in Fig. 1a. These populations generally comprise of an innermost necrotic zone, a middle quiescent layer, where the cells are viable but not growing, and an outer proliferative layer that leads to most of the tumour growth (Hirschhaeuser et al. 2010). However, while 3D multicellular spheroids have proven to be a better model to mimic an in vivo tumour, experimentation in furthering cancer treatments has not been widely explored.

When evaluating the effect of radiotherapy (RT) on the survival of cancer cells, 3D spheroids are a more viable option compared to the 2D monolayer as they are more representative of an actual in vivo TME. There is an observed increase in resistance to RT when comparing 3D spheroids to 2D culture (Brüningk et al. 2020). Furthermore, while the use of RT is widely used in the clinic, the benefits it provides to treating tumours have been largely optimized by modalities, such as image guided RT (IGRT) for precise beam positioning and intensity modulated RT (IMRT) for dose conformity (Otto 2008; Xing et al. 2006). Thus, to improve patient outcomes, novel techniques must be explored, such as the use of nanomaterials. One nanomaterial—gold nanoparticles (GNP)—offer many potential applications in cancer therapy due to its high density (*Z* = 79) compared to soft tissue (*Z* $\approx$ 7.5). GNPs can be used to enhance the effectiveness of radiation treatment by increasing the absorption of radiation in cancer cells. When GNPs are exposed to ionizing radiation, such as X-rays or gamma rays, they can generate cascades of electrons that can cause damage and produce reactive oxygen species that can kill cancer cells. This process, known as the GNP-mediated radiosensitization effect, has been shown to be effective in preclinical studies using animal models (Choi et al. 2018). The uptake dynamics of GNPs in 2D monolayer and 3D spheroids has previously been optimized, using various cell lines (Bromma et al. 2020, 2021, 2022).

To further improve the combined treatment of RT and GNPs, we can also include the chemotherapeutic agent docetaxel (DTX). DTX is a commonly used therapeutic agent that is used to treat several cancers, including cervical cancer and prostate cancer (Pearl et al. 2007; Puente et al. 2017). DTX works by causing the unregulated formation of microtubules (Pienta 2001). Furthermore, DTX can also act as a radiosensitizer due to its ability to arrest the cell cycle and synchronize a population into the radiosensitive G2/M phase. During cell division, a cell will go through mitosis and form a mitotic spindle. However, when treated with DTX, the microtubules are sequestered into bundles and thus are hindered in the formation of the mitotic spindle (Paoletti et al. 1997). DTX also forms asters and bundles independent of centrosomes which can allow for multiple cleavage planes, leading to a mitotic catastrophe. This results in cells that are either stuck in mitosis or form a multi-nucleated cell as the nuclear envelope surrounds the multiple asters. Thus, when combining DTX with GNPs and RT, we expect to see an additional radiosensitizing effect beyond treatment with GNPs and RT alone. The use of DTX with GNPs has also been shown to improve long term cellular retention, a benefit when it comes fractionated therapy (Bannister et al. 2020b). The benefit of using this combined therapy can be seen in Fig. 1b, where the utilization of GNPs and DTX adds two radiosensitizers that can stimulate further damage in the tumour periphery with radiation, leading to improved cell kill and a reduced growth response. By more effectively removing the outer cells of the spheroid, a tumour can be ‘peeled’ like an onion with multiple fractions, leading to eventual death of the cancer.

This study’s goal was to explore the application of GNPs and DTX in a combined therapy modality with RT. This response will be compared in a 2D monolayer model and 3D spheroid model to showcase the benefit of using GNP and DTX even if the penetration depth of the combination treatment follows the concentration gradient. Furthermore, this work can be used to compare in future work in in vivo animal models to observe any limitations in dose response. This is one of the final pillars in the foundation for a better platform for testing GNPs in the lab before entering the clinic.

## Results and discussion

### Characterization of gold nanoparticles and spheroids

For this study, small spherical GNPs of approximately 17 nm in diameter. The uptake of GNPs depends largely on their size, with larger GNPs of size ~ 50 nm being more efficiently taken up by cells in monolayer (Chithrani et al. 2006). However, smaller GNPs will better be able to penetrate the dense ECM towards deeper cells in spheroids (Bromma et al. 2021). These GNPs were conjugated with polyethylene glycol (PEG) and a peptide containing integrin binding domain RGD. The addition of PEG ensures stability in serum, such as in vivo circulation, while the addition of the RGD peptide improves uptake and can target integrin-overexpressing cells, commonly found in cancer (Manson et al. 2011; Wu et al. 2017). Furthermore, the use of RGD is also beneficial in targeting the ECM. The RGD motif is expressed within various proteins, such as fibronectin, vitronectin, fibrinogen, osteopontin, and bone sialoprotein (Bellis 2011).

**Fig. 2:**
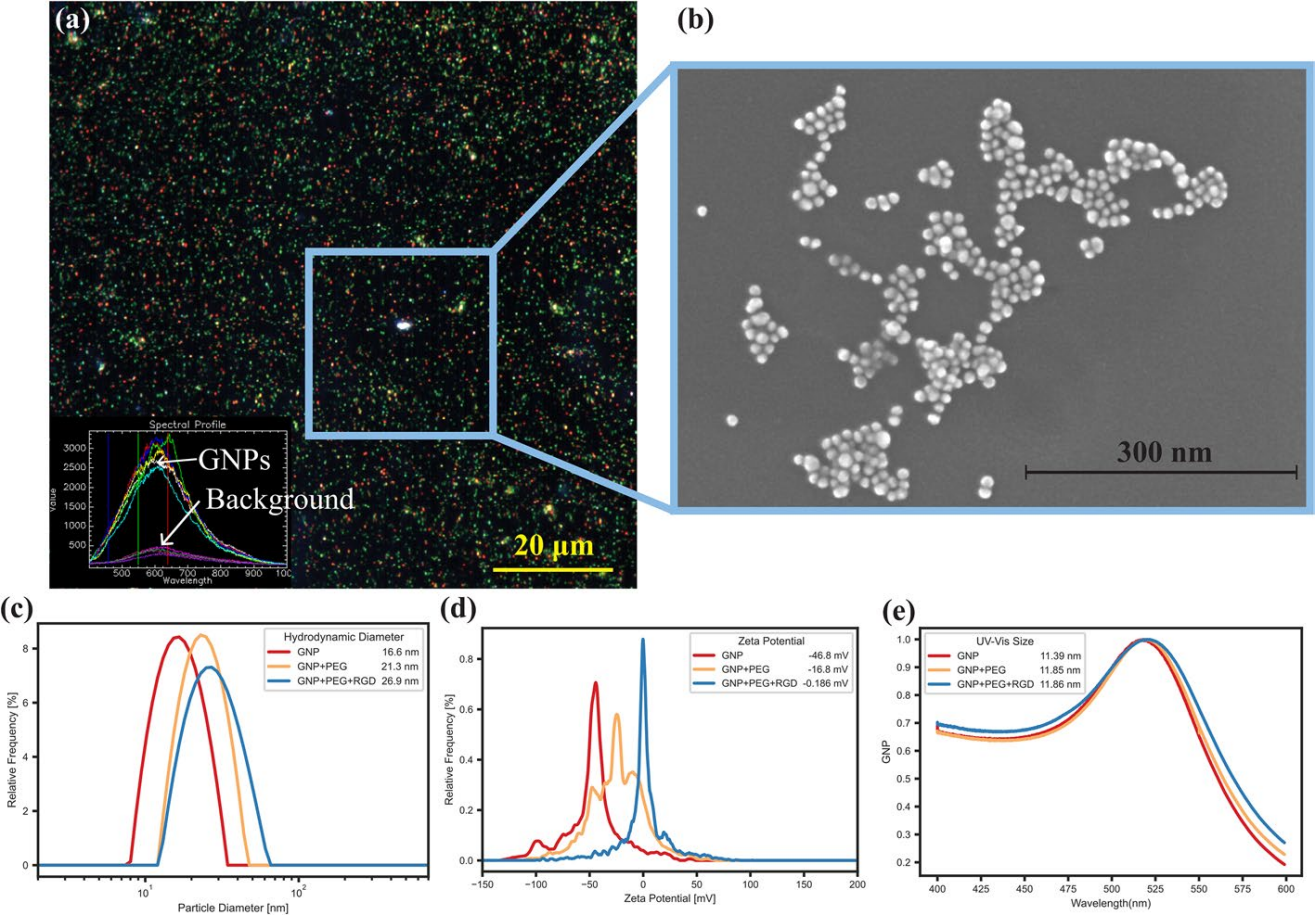
Characterization of gold nanoparticles. **a** Hyper spectral imaging of gold nanoparticles decorated with polyethylene glycol (PEG) and a peptide containing RGD. Scale bar is 20 $\upmu$m. Inset is hyper spectral spectrum of gold nanoparticles and background. **b** Secondary electron image from a transmission electron microscope of gold nanoparticles. Scale bar is 300 nm. **c** Size of gold nanoparticles measured by dynamic light scattering while bare, with PEG, and with PEG and RGD. **d** Surface $\upzeta$-potential of bare and decorated GNPs. **e** Size as measured by the UV–visible absorbance spectrum of bare and decorated GNPs

Smaller nanoparticles have been found to enter cancer cells more effectively in more complex spheroid environments as well as in an in vivo environment (Cho et al. 2010). This is a result of the ECM limiting the penetration depth of larger NPs. Visualization of the GNP complexes can be done using hyper spectral imaging (HSI), as shown in Fig. 2a, to gather their spectrum for future verification in cells. Verification of GNP presence using HSI has been widely used in literature, from monolayer to in vivo section analysis (Zamora-Perez et al. 2018). HSI is a useful technique as it allows for each pixel in an image to be associated with a complete reflectance spectral response, with a spectral resolution of 2 nm in the visible–near-infrared range. GNPs have a unique reflectance spectral response, due to their surface plasmon resonance (Amendola and Meneghetti 2009). Thus, using HSI allows us to capture this response and easily identify GNPs compared to tissue or cells. The GNP complexes were also measured using scanning transmission electron microscopy (STEM; Fig. 2b) to verify GNP size, shape, and stability following functionalization. The measured average core size was $12.47\pm 1.21\mathrm{ nm}$ conjugation with PEG and RGD. Bare GNPs had a similar measured core size of $12.76\pm 2.8 \mathrm{nm}$, signifying no aggregation was present. Measurement of the GNPs before and after conjugation with PEG and RGD was also done using dynamic light scattering (DLS; Fig. 2c). DLS measured the bare GNPs to have a hydrodynamic diameter of $16.6\pm 0.33\mathrm{ nm}$, $21.3\pm 0.43 \mathrm{nm}$ after PEGylation, and $26.9\pm 0.54 \mathrm{nm}$ after addition of RGD peptide The combination of GNP, PEG, and RGD is referred to as a GNP complex throughout this work. The difference in the size of the measured diameter between DLS and measurement using STEM is due to the DLS measuring the hydrodynamic diameter, which includes surface ligands. STEM images only show the dense gold in the nanoparticle core (Wang et al. 2019). The size of the GNPs was also measured using ultraviolet–visible (UV–Vis) spectrometry to estimate the size and concentration while confirming stability of the GNP complex (Fig. 2e) (Haiss et al. 2007). The spectral response, specifically the ratio of the peak wavelength absorption to the absorption at 450 nm, allows an approximate measurement of the size and concentration of the GNPs. The measured size was found to increase from 1 $1.4\pm 0.1 \mathrm{nm}$ to $11.9\pm 0.1 \mathrm{nm}$ following functionalization with PEG and RGD, further indicating no aggregation and a stable formulation.

The $\upzeta$-potential is a useful measure of the suspension stability and can be used to confirm surface adsorption due to different electric potentials of various moieties (Xu 2008). To verify conjugation of PEG and RGD, the $\upzeta$-potential (Fig. 2d) was measured to be $-46.8\pm 1.24\mathrm{ mV}$ for bare GNPs, $-16.80\pm 0.63\mathrm{ mV}$ for PEGylated GNPs, and $-0.19\pm 0.28 \mathrm{mV}$ for GNP complexes. The increase in the $\upzeta$-potential is due to the replacement of the negatively charged citrate molecules on the surface of GNPs with neutral PEG molecules and positively charged RGD peptides. It has been previously shown that the GNP complexes are stable in highly ionic environments (Bromma et al. 2022). Furthermore, as can be seen in Additional file 1: Fig. S1c, the GNP complexes are stable after storage for 2 months at 4 °C. Thus, these GNP complexes are suitable for testing purposes.

To optimize the treatment with GNPs, the 3D spheroids were characterized. While a 2D model involves single cells, the 3D spheroids introduce a more complex environment, including the ECM and a drug concentration gradient, as shown in Fig. 1. For the 3D spheroid, a diameter of approximately 300–400 $\mathrm{\mu m}$ was chosen as an optimum spheroid size. Larger spheroid sizes (> 350 $\mathrm{\mu m}$) can introduce a necrotic core and increased number of quiescent cells, affecting results (Singh et al. 2020). Different cell lines will have different packing densities and thus will require a varying number of initial cells for spheroid formation. It was observed that 3125 cells for HeLa and 1000 cells for LNCaP was optimal, as seen in Additional file 1: Fig. S1a, b. The packing density has been previously shown to affect the efficacy and penetration of anticancer drugs and smaller nanoparticles. Mimicking these in vivo effects is one of the many benefits of using a 3D spheroid model over a 2D monolayer model (Grantab et al. 2006).

### Docetaxel in spheroids and monolayers

**Fig. 3:**
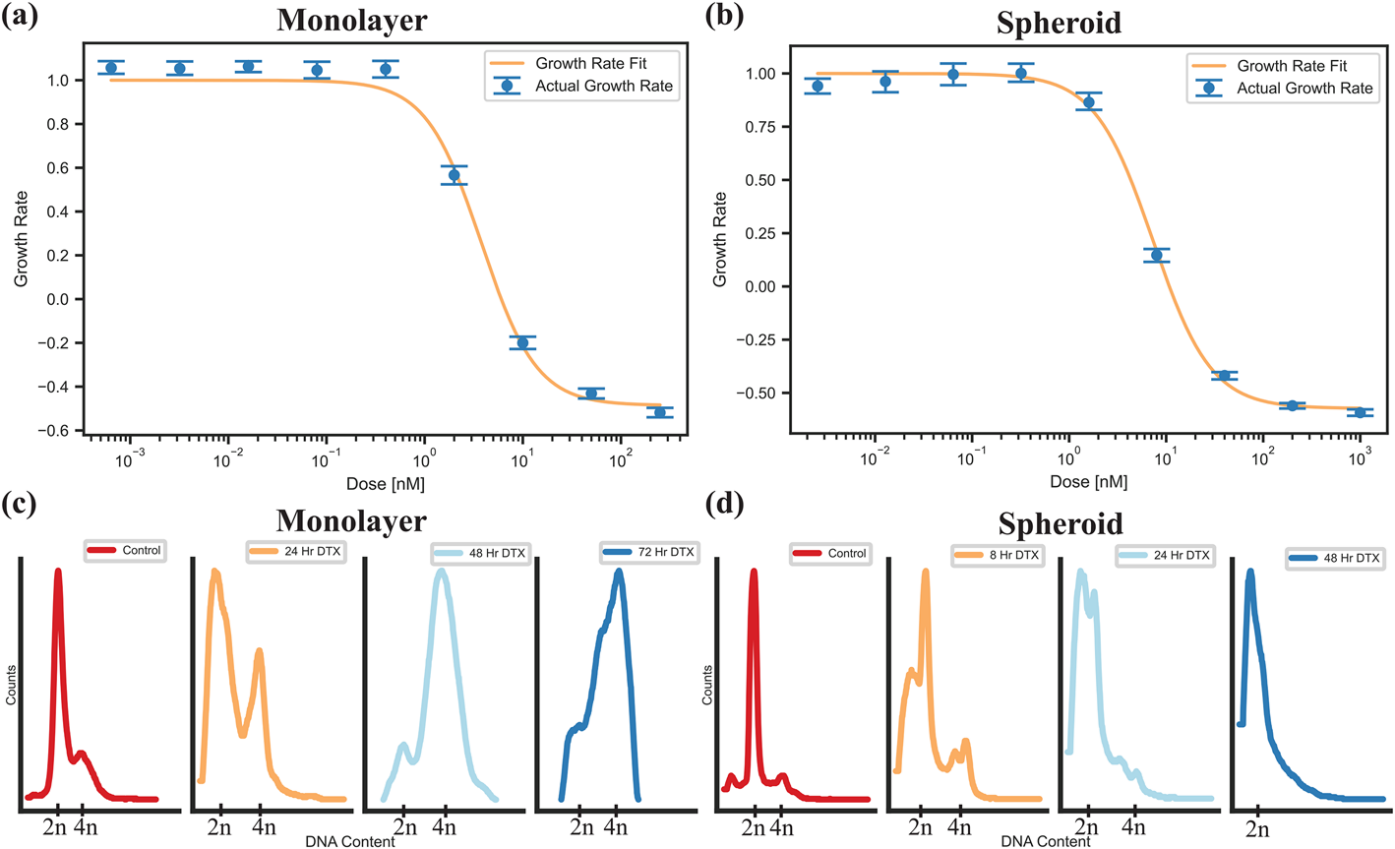
Docetaxel characterization in HeLa. **a**, **b** Proliferation assays for a (**a**) two-dimensional monolayer and a (**b**) three-dimensional spheroid, for the cervical cancer cell line HeLa, treated with docetaxel. **c**, **d** Cell cycle analysis of a (**c**) monolayer and (**d**) spheroids of HeLa cells treated with the GR50 dose of docetaxel, calculated per modality

DTX has been shown to synchronize cells in the G2/M phase of the cell cycle, thus effectively acting as a radiosensitizer. Furthermore, DTX can also alter the uptake of GNPs in monolayer as well as spheroids (Bromma et al. 2020). Due to toxicity of DTX at higher doses, the ideal dose will not increase cell death, but rather synchronize the cell population. This will effectively improve the uptake of GNPs and act synergistically with the radiosensitizing effects of the GNP complexes. To measure the optimal dose, we used the growth rate inhibition metric to observe the dose in which division time was essentially halted and cytostasis was achieved (Hafner et al. 2016). For monolayer, this occurred at $3.78\pm 0.16$ nM and $0.94\pm 0.034$ nM for HeLa and LNCaP, respectively (Fig. 3a). These doses agree with IC50 values found in literature. For spheroids, HeLa needed a dose of $7.33\pm 0.25$ nM and LNCaP required a dose of $3.93\pm 0.50$ nM (Fig. 3b). The larger DTX dose compared to monolayer is a result of the higher resistance to treatment inherent to the more complex 3D environment. Proliferation data for LNCaP can be seen in Additional file 1: Fig. S2a, b. Compared to a clinical dose, the area under concentration–time curve (${\mathrm{AUC}}_{0\to 24}$) is $90.70\pm 3.84 \mathrm{nM}\cdot \mathrm{h}$ and $22.56\pm 0.82\mathrm{ nM}\cdot \mathrm{h}$ for a monolayer of HeLa and LNCaP, respectively. For HeLa and LNCaP spheroids, respectively, the ${\mathrm{AUC}}_{0\to 24}$ was $175.92\pm 6.00 \mathrm{nM}\cdot \mathrm{h}$ and $94.32\pm 12.00 \mathrm{nM}\cdot \mathrm{h}$ for. A median ${\mathrm{AUC}}_{0\to 24}$ of 1284 nM $\cdot$ hr is observed clinically for a lower dose of 20 mg/${\mathrm{m}}^{2},$ showing the implemented doses are clinically comparable. Furthermore, the doses tested will have low concerns of potential side effects when translating to a clinical environment (Fr Brunsvig et al. 2007).

The synchronization effects of DTX in monolayer and in spheroids were then explored using flow cytometry (Fig. 3c, d). DTX has previously been shown to synchronize cell populations in the radiosensitive G2/M phase of the cell cycle. For a monolayer of HeLa, there was a fast shift from a normal cell population to a large population of cells in the G2 phase after 24 h. The shift to a G2-synchronized cell population happened more gradually for spheroids. These results indicate the chosen dose is working as desired. The inability to differentiate between G1/G2 peaks in the spheroids may be indicative of mitotic catastrophe, where the cells become multinucleated and only have single pairs of DNA or less per nucleus. However, they are still synchronized. This synchronization effect was maintained for at least 72 h for both cell models. For LNCaP (Additional file 1: Fig. S2c, d), we did not see a synchronization effect in monolayer or spheroids. This is indicative of either a resistance in the LNCaP cells to DTX or cells that were affected by DTX failed to transition through S phase to the G2 phase. This effect has previously been observed in literature (Lee and Lee 2021).

The cell response due to the desired doses of DTX were explored using darkfield imaging. DTX causes unregulated formations of microtubules, sequestering them into bundles which hinders the formation of the mitotic spindle necessary for cell division (Paoletti et al. 1997). As a result, in a monolayer of HeLa cells, compared to control (Additional file 1: Fig. S3a, b), we can see some multinucleated cells caused by this mechanism of action in the darkfield images. A multinucleated cell is circled in red in Additional file 1: Fig. S3b. Spheroids appear to have a slightly disrupted core compared to untreated spheroids (Additional file 1: Fig. S3c, d). However, the extent is limited, allowing for the bulk of the spheroid to maintain a spherical structure. Hyper spectral profiles, from HSI microscopy, of the cells and background were added to each darkfield image as an inset figure to verify the presence or absence of measured GNPs in the image. The lines in the image are the mapping of what wavelength corresponds to blue, green, and red for the displayed hyper spectral image.

To compare the in vitro 2D and 3D models, we measured the GNP uptake in monolayer as well as in spheroids after treatment with DTX. Both models were treated with GNP complex at a clinically relevant dose of 10 $\upmu$g/mL for 24 h. This dose has shown radiosensitization effects before at megavoltage (MV) energy ranges in a prostate cancer in vivo model (Wolfe et al. 2015). We can see in Additional file 1: Fig. S3a the measured uptake of the GNP complex in monolayer for both cell lines for control and DTX. There was a 15% increase for HeLa which was not significant, while in LNCaP, there was a significant increase in uptake of 75.4% (*p* < 0.05). While LNCaP did not have observed synchronization of the cells, there was still an effect that increased the GNP uptake in both cell lines.

**Fig. 4:**
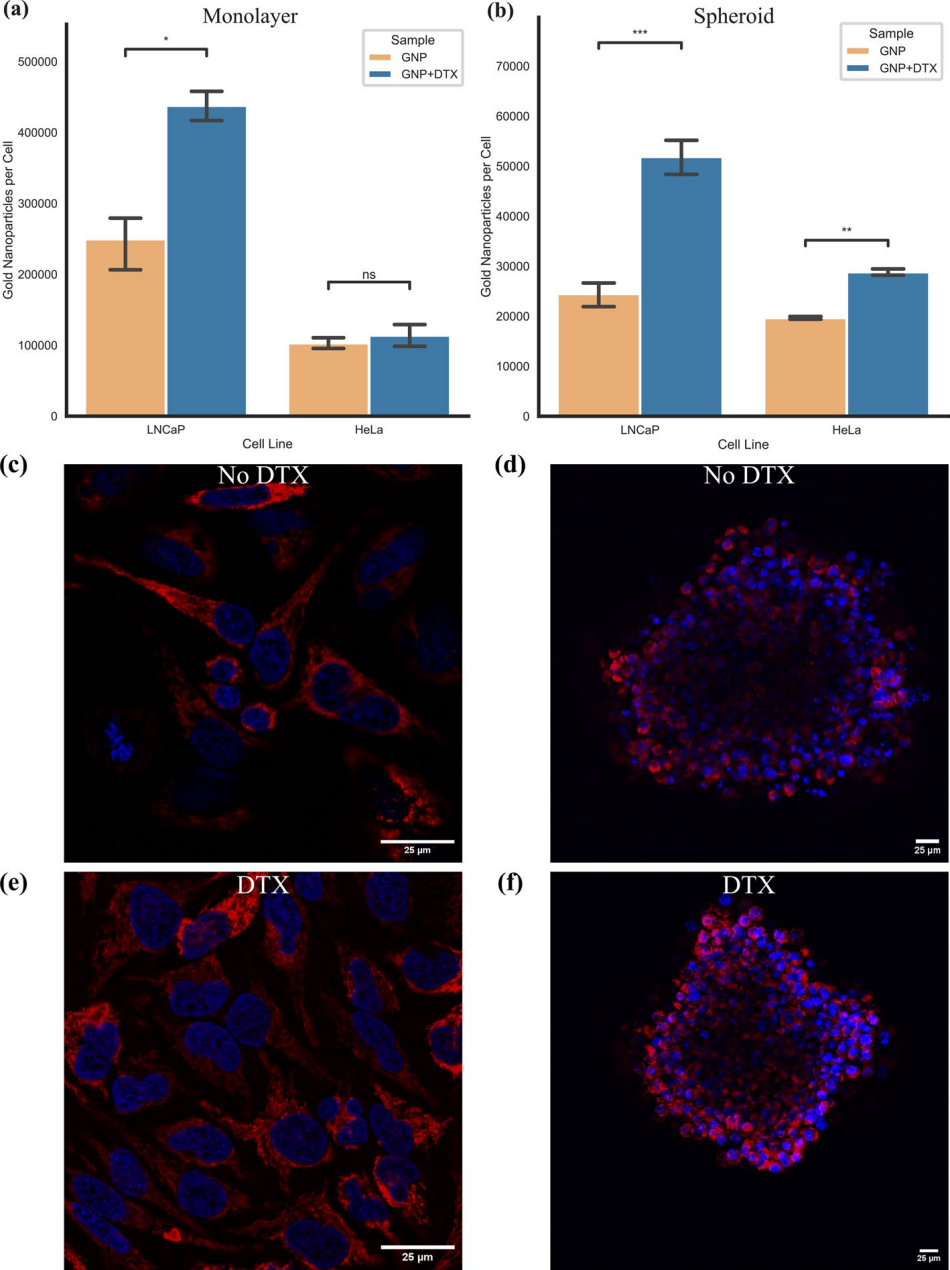
Gold nanoparticle uptake in monolayer and spheroid. **a**, **b** Uptake of gold nanoparticles in **a** monolayer and **b** spheroids of HeLa and LNCaP with and without docetaxel. Error bars are the 95% confidence interval from three independent experiments. **c**, **e** Confocal images of a monolayer of HeLa with gold nanoparticles, **c** without and **e** with docetaxel. **d**, **f** Confocal images of spheroids of HeLa with gold nanoparticles, **d** without and **f** with docetaxel. Gold nanoparticles and nuclei are marked in red and blue, respectively. Scale bars are 25 $\upmu$m. ns indicates no significance, * indicates 0.01 < *p* < 0.05, ** indicates 0.001 < *p* < 0.01, *** indicates 0.0001 < *p* < 0.001

In Fig. 4, the measured uptake of GNP complex in spheroids for both cell lines, with and without treatment of DTX, is displayed. There was a significant increase in uptake after dosing with DTX of 46.57% (*p* < 0.001) and 111.9% (*p* < 0.001) in both HeLa and LNCaP spheroids, respectively. Both HeLa and LNCaP saw beneficial uptake in monolayer and in spheroids with the addition of DTX, with the increase in spheroids being more drastic. This effect is observed despite the dose of DTX being normalized to the same growth rate. This may be a result of a higher dose of DTX in spheroids that will disproportionately affect the outer cells. The outer cells are the same cells that will have the largest uptake of GNPs, due to the concentration gradient. These cells will experience a larger DTX effect due to the higher dose and have an amplified uptake compared to monolayer. In monolayer, there is a lower dose of DTX and thus less effect on the cells. This increased effect agrees with previously measured values on separate cell lines from prior experiments (Bromma et al. 2021; Bromma et al. 2020). Due to the synchronization of the cells into the G2/M phase with DTX, it is expected for a higher quantity of GNPs to be present in the cell population relative to other phases. This is a result of having a larger time in the cell cycle to accumulate GNPs before division. The process of cell division will lead to the dilution of the per-cell GNP load, observed in cells that are distributed in the cell cycle more regularly.

For the HeLa cancer cells, after concomitant treatment of DTX and GNP complex, we can see in confocal images the accumulated GNPs in monolayer (Fig. 4c, e) and in spheroids (Fig. 4d, f) vs control. The GNPs can be visually seen to increase in the monolayer of cells, despite the quantification not having an observable statistically significant increase in uptake. We have also previously shown that DTX-treated cells tend to gather the GNPs closer to the nucleus, which may result in an increased radiosensitization effect to the most vulnerable organelle (Bannister et al. 2020a). Furthermore, we can see how the distribution of GNPs is largely contained to the edge of the spheroid in the confocal images of the spheroids (Fig. 4d, f). In the DTX-treated sample, the spheroid is slightly smaller, possibly due to halting of the cell cycle while also having a visual increase in the amount of gold. The increased GNP uptake in the shell of the spheroid can allow for an increased efficacy in a fractionated treatment. This can more effectively ‘peel the onion’ of the tumour layer by layer—or fraction by fraction—due to the increased radiosensitization caused by GNPs.

### Radiation response in monolayer

The gold standard for measuring cell reproductive death following treatment with radiation is the clonogenic assay. However, while the clonogenic assay is effective, it is susceptible to human error and inconsistencies. The application of a proliferation assay can measure viable cells over time effectively and with very little error due to larger cell counts. In addition, a 2D-based assay such as the clonogenic assay that is applicable to a more homogeneous population is not the most optimum approach, due to the heterogeneous nature of 3D structures. Thus, for a monolayer of HeLa and LNCaP, the use of the PrestoBlue assay was applied to measure radiation efficacy. PrestoBlue is a resazurin-based reagent that, in the presence of various proteins in the cell that are relevant in cellular respiration, such as FADH and NADPH, will reduce to resorufin (Xu et al. 2015). Resorufin is strongly fluorescing and is an accurate measurement of total cell viability. Thus, following radiation, we can use PrestoBlue to measure survival of the cellular population under different treatments.

**Fig. 5:**
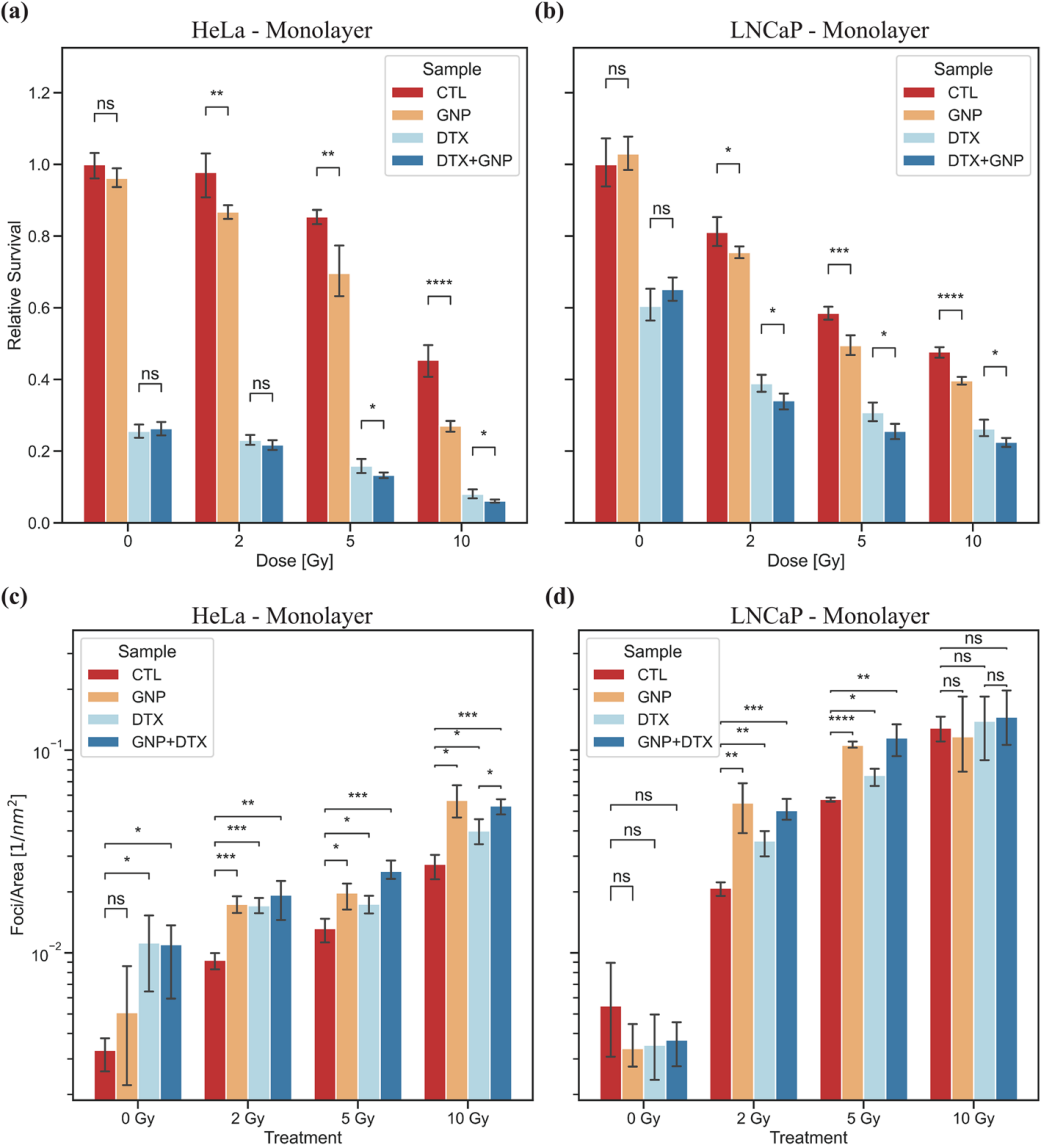
Radiation response in monolayer. **a**, **b** Normalized PrestoBlue proliferation assay after 6 days following radiation for **a** HeLa and **b** LNCaP. Error bars are the 95% confidence interval from three independent experiments. Cells were irradiated with 0, 2, 5, and 10 Gy using a 6 MV linear accelerator. **c**, **d** Normalized 53BP1 foci per nuclear area 24 h following radiation in **c** HeLa and **b** LNCaP. Error bars are the 95% confidence interval from at least 50 nuclei from three experiments. ns indicates no significance, * indicates 0.01 < *p* < 0.05, ** indicates 0.001 < *p* < 0.01, *** indicates 0.0001 < *p*< 0.001, **** indicates *p* < 0.0001

Measurement of monolayer growth using PrestoBlue was limited to 6 days, as the populations reached confluence past this point. Following 24 h of treatment with GNPs and DTX, the cells were either not radiated, or radiated with 2 Gy, 5 Gy, and 10 Gy. The survival data after 6 days can be seen Fig. 5a, b. We can observe that for unirradiated HeLa samples (Fig. 5a), DTX limited the growth in monolayer effectively. Moreover, for 0 Gy, there was no significant difference when adding GNPs to either untreated samples or samples treated with DTX. This highlights the safety of these GNP complexes. When the cells are irradiated with 2 Gy, we can see that there is a significant difference in survival between control and GNP-treated samples Survival lowers from 97.82% to 86.79%, a relative change of 11.29% (*p* < 0.001). There was no significant difference in DTX treated samples. In 5 Gy and 10 Gy samples, we see a similar trend in difference in survival between control and GNP-treated samples. After 5 Gy, there is a reduction in survival from 85.40% to 69.62%, a relative change of 18.48% (*p* < 0.001). For 10 Gy, survival reduces from 45.45% to 27.02%, a relative change of 40.55% (*p* < 0.00001). Furthermore, in DTX-treated and combined GNP + DTX-treated samples, we see a significant decrease in survival after radiation with 5 Gy and 10 Gy. For 5 Gy, DTX samples had a survival of 15.84% and DTX + GNP samples had a survival of 13.27%, a relative change of 16.22% (*p* < 0.05). For 10 Gy, DTX samples had a survival of 8.04% and DTX + GNP samples had a survival of 6.09%, a relative change of 24.25% (*p* < 0.05). LNCaP (Fig. 5b) had a similar result.

There is an observed trend of a larger relative decrease in survival in GNP-treated samples when the dose of radiation increases. This observation makes sense when recalling the mechanism of action of GNPs. As more high energy X-rays interact with the GNPs, there will be a larger amount of energy absorbed in the gold relative to soft tissue. This will lead to increased cascades of Auger electrons, due to an increase in the photoelectric effect. There will thus be an increase in ROS production, because of the flux of secondary electrons, which leads to an elevated radiosensitizing effect (Butterworth et al. 2012). As a result, there will be an increase in damage to DNA and cellular structures. With larger doses, this effect will be amplified as the repair abilities in the cell will be overloaded due to the increase in damage. When adding DTX, there is a further increase in radiosensitizing effect. DTX's radiosensitizing ability is accomplished through cell cycle synchronization in the G2/M phase. As there is no increase in secondary electrons or ROS production, the effect of DTX should be relatively consistent as the radiation dose increases. When adding GNPs to DTX-treated samples, we observe a similar decrease in survival with increasing radiation dose. Overall, in monolayer, there appears to be a benefit in using GNPs with higher doses. This may be especially useful in prostate cancer, as many protocols that treat these malignancies require hypo-fractioned dose regimes (Hickey et al. 2019).

We performed an immunofluorescent assay to further validate the data generated from cell proliferation curves. This was done by probing DNA damage using antibodies against the repair protein 53BP1. This protein is present in the event of a DNA DSB. The 53BP1 foci were measured 24 h after treatment which allows us to gather information of residual damage. The remaining DNA damage is likely to go unrepaired and is more likely to lead to loss of clonogenic potential (Banáth et al. 2010). The number of foci can depend on the type of damage as well as the damage repair mechanism. The 53BP1 foci signal non-homologous end joining (NHEJ) as a repair mechanism, while a lack of the 53BP1 but presence of a $\upgamma$ H2AX foci could signal homologous repair in the S or G2 phase (Popp et al. 2017). This could lead to a varying number of $\upgamma$ H2AX foci compared 53BP1 foci. Further to this point, during S phase, there is a pan-nuclear $\upgamma$ H2AX speckle that can form, leading to miscounting of these foci. Meanwhile, during the S-phase, 53BP1 can still act, via NHEJ, in a H2AX independent manor, suggesting that 53BP1 can function in DNA repair independently of H2AX (Yuan et al. 2010). High expression of $\upgamma$ H2AX can also be suggestive of defective DNA repair pathway and/or genomic instability, whereas 53BP1 is a conserved checkpoint protein DNA DSB sensor. Confocal images of HeLa and LNCaP showcasing 53BP1 foci (green) in the nuclei (blue) can be seen in Additional file 1: Figs. S4 and S5, respectively.

Furthermore, the number of foci, which are a proxy for DNA DSBs, per nuclear area was quantified. For HeLa, after no radiation, we observe that DTX had a significant increase in foci/area (DTX: *p* < 0.05, GNP + DTX: *p* < 0.05) relative to control. DTX is not known to increase production of DNA DSBs by itself, so this result could simply be due to radiosensitization caused by DTX. This increase in radiosensitivity can then allow the background radiation to cause more damage in the genomically unstable HeLa cells (Frattini et al. 2015). This same effect was not observed in LNCaP. This is likely due to LNCAP having a lower response to DTX as well as being more genetically stable (Greene et al. 2016).

When looking at irradiated HeLa and LNCaP, there is a significant increase in 53BP1 foci when treated with GNPs, DTX, and combined GNP/DTX. For LNCaP, after being radiated with 10 Gy, the number of 53BP1 foci was saturated and counting the increase of individual foci was rendered difficult. As a result, at 10 Gy, LNCaP had no significant result. As expected, the base level—without GNPs and DTX—of 53BP1 foci per area increased with increasing radiation dose. Following radiation for both HeLa and LNCaP, the increase of 53BP1 foci can be observed in both GNP and GNP + DTX treated samples, compared to control and just DTX. For HeLa, compared to control after irradiation with 2 Gy, we see an increase in DNA DSB foci per nuclear area of 87.9% (*p* < 0.001), 85.7% (*p* < 0.001), and 109.9% (*p* < 0.01) for GNP, DTX, and GNP + DTX treated samples, respectively. Likewise, for 5 Gy, we see an increase of 49.6% (*p* < 0.05), 31.9% (*p* < 0.05), and 91.6% (*p* < 0.001) for GNP, DTX, and GNP + DTX treated samples, respectively. Finally, for a 10 Gy dose, we see an increase in foci of 107.0% (*p* < 0.05), 46.1% (*p* < 0.05), and 94.3% (*p* < 0.001) in GNP, DTX, and GNP + DTX treated samples, respectively. Similar results were observed for LNCaP, except when signal was saturated at 10 Gy.

We have previously shown that GNPs can increase the number of foci present in a sample following 2 Gy of radiation (Bromma et al. 2020). This is consistent with this result while also showing that higher doses of radiation lead to an increase in DSBs compared to DTX and control. Furthermore, LNCaP has a larger relative increase in DNA DSBs when irradiated with any dose, indicating that LNCaP in monolayer has a larger DNA damage response than HeLa.

### Radiation response in spheroids

Exploration of the trends observed in a 2D monolayer and if they will stay consistent in a 3D spheroid will help elucidate the benefits of both models. As seen in Additional file 1: Fig. S3, there will be a differential amount of uptake of GNPs in spheroids when treated with DTX, compared to monolayer. However, to ensure the spheroids we are testing have normoxic conditions, we qualitatively measured the hypoxia present in the core of the cells. It is important to ascertain the approximate oxygen levels, as hypoxia is one of the most important factors reducing RT effectiveness (Horsman et al. 2012). To accomplish this, we used Image-iT Hypoxia Reagent, which fluoresces in states of low oxygen. In spheroids, this would occur naturally within the core when they grow too large, due to the concentration gradient. In Additional file 1: Fig. S6 we measured spheroids of diameter ranging from 200 $\upmu$m up to 1000 $\upmu$m. For the spheroids used in our experiments, which have a diameter of approximately 300–400 $\upmu$m, there was no visible fluorescence and thus no measured hypoxia. However, once spheroids were sizes larger than about 600 $\upmu$m, fluorescence started being observed, signifying a lack of ${\mathrm{O}}_{2}$ and a resulting formation of a hypoxic core.

**Fig. 6:**
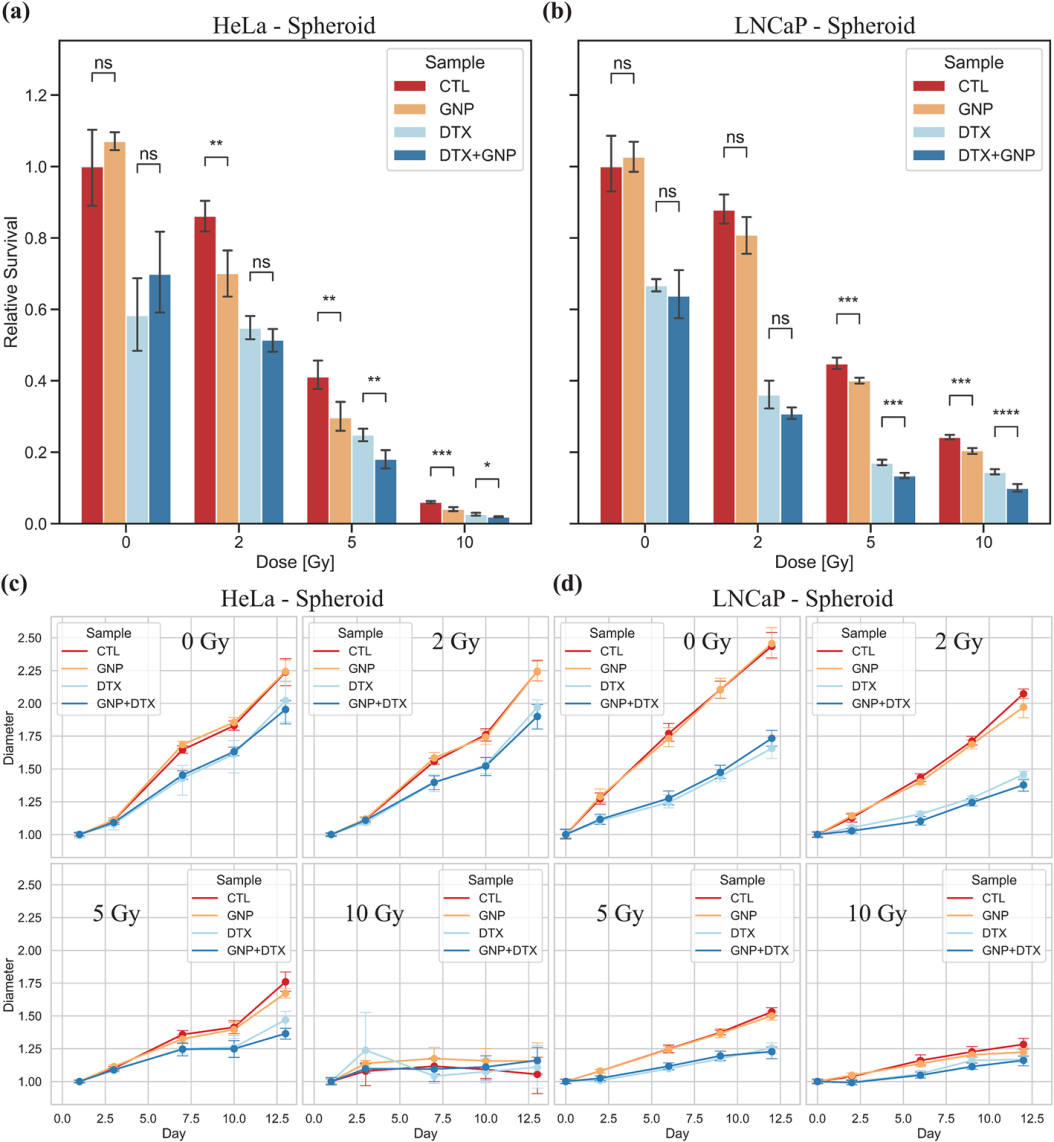
Radiation response in spheroids. **a**, **b** Normalized CellTiter-Glo 3D cell viability assay after 13 days following radiation for spheroids of **a** HeLa and **b** LNCaP. **c**, **d** Normalized increase of diameter relative to starting size of **c** HeLa spheroids and **d** LNCaP spheroids over 13 days following radiation. Error bars are the 95% confidence interval from three independent experiments with at least 5 repeats per condition per experiment. ns indicates no significance, * indicates 0.01 < *p* < 0.05, ** indicates 0.001 < *p* < 0.01, *** indicates 0.0001 < *p* < 0.001, **** indicates *p* < 0.0001

As the spheroids we are using are all normoxic, there should be no reduction in effect from radiation due to lack of ROS production. To measure the effect radiation had on the spheroids, we measured the survival of the spheroids after 14 days using a luminescent 3D-based cell viability assay. Like monolayer, following 24 h of treatment with GNPs and DTX, the HeLa and LNCaP spheroids were either unirradiated, or radiated with 2 Gy, 5 Gy, and 10 Gy. As shown in Fig. 6a, b, there was no significant difference in survival in non-irradiated samples when treated with GNPs. This further demonstrates the safety of the current GNP complexes after a longer time span of 14 days. Moreover, when HeLa spheroids are treated with GNPs and 2 Gy of radiation, there is a significant decrease in survival from 86.18% to 70.12%, a relative change of 18.6% (*p* < 0.05). With DTX treated HeLa spheroids, this does not translate to a significantly different result after 2 Gy. For all 2 Gy treated LNCaP spheroids, there is no statistically significant change in survival, though there is a trend of more damage with GNPs in both control and DTX treated samples.

When we move up to higher radiation doses of 5 Gy and 10 Gy, a trend of increased damage due to the addition of GNPs can be seen. After irradiating HeLa spheroids with 5 Gy (Fig. 6a), there is a relative decrease in survival of 27.74% between control and GNP samples, from 41.14% to 29.7% (*p* < 0.01). Between DTX and DTX + GNPs treated samples, there is a relative decrease in survival of 16.55%, from 24.87% to 18.07% (*p* < 0.01). Likewise, for HeLa treated with 10 Gy, there is a relative change of 32.33%, from 6.01% survival to 4.06% for control and GNP (*p* < 0.001). Finally, for DTX and DTX + GNPs samples irradiated with 10 Gy, there was a 27.85% relative reduction from 2.65% to 1.92% (*p* < 0.05) survival.

We see a similar trend in LNCaP irradiated with 5 Gy and 10 Gy. For LNCaP and 5 Gy (Fig. 6b), we see a reduction in survival from 44.82% for control to 40.08% for GNPs, a relative change of 10.54% (*p* < 0.001). Furthermore, there is reduction in survival from 17.01% to 13.45% for DTX vs DTX + GNP samples, a relative change of 20.90% (*p* < 0.001). For 10 Gy, there is a similar effect, with a relative change of 15.61% when comparing control (24.22%) and GNP-treated (20.44%) samples (*p* < 0.001). Finally, when comparing DTX and DTX + GNPs for LNCaP with 10 Gy, there is a relative change of 31.04%, with DTX having 14.43% survival, and DTX + GNP having 9.95% survival (*p* < 0.0001).

Unlike the DSB measurements in monolayer, we can see that HeLa is more radiosensitive compared to LNCaP and will naturally have a lower survival as radiation doses increase. For both cell lines in both monolayer and spheroids, it appears that a dose of 2 Gy is not enough to elicit a large radiosensitization effect. However, there is a trend pointing towards more damage overall with GNPs added to treatment. When moving into higher doses, such as 5 Gy and 10 Gy, there is a significant decrease in survival of the cancer cells due to the addition of GNPs, both in spheroid and in monolayer. For LNCaP spheroids, this effect seems to be consistently around $\approx 10$–$15$% when not treated with DTX, and $\approx 20$–$30$% when treated with DTX. This suggests that for LNCaP, the addition of DTX and GNPs as a combined therapy works synergistically to improve the radiosensitization effect. Interestingly for HeLa spheroids, when treating at higher doses, this does not seem to be the case. While there is an increase in damage when adding GNPs with DTX, it does not lead to a larger relative change than in control with GNPs without DTX. There is a general change of $\approx 16$–$32$% when GNPs are added overall in HeLa for higher doses.

One limitation of using the CellTiter-Glo 3D assay for spheroids as well as the PrestoBlue resazurin-based assay for monolayer is the measurement of quiescent cells in the cancer population. This will be more pronounced for spheroids, as they tend to form a quiescent mid-layer, as described in Fig. 1a, as they grow. Moreover, with the addition of cytotoxic drugs, such as DTX and radiation, there will be an increase of senescent cells. These cells are seriously damaged but not dead. Therefore, they will still add to the signal due to metabolic activity. However, highly utilized alternatives such as a clonogenic assay are not really viable for a spheroid, due to the many layers that include necrotic and senescent cells that will lead to a heterogeneous population. Furthermore, clonogenic assay does not account for how spheroids will be structurally altered as growth occurs following treatment. Thus, despite the limitations of viability-based assays, such as CellTiter-Glo 3D, it allows for the spheroids to maintain their structure until measurement. This leads to a more accurate representation of the state of the cancer.

We can use the growth curves (Fig. 6c, d) to visualize the relative change in diameter of the spheroids over approximately 2 weeks following treatment. As expected, with more radiation, there was a decrease in visual spheroid growth with an increase in radiation and with treatment of DTX. DTX will naturally slow the growth, due to blockade of the cells into G2/M phase of the cell cycle (Hernández-Vargas et al. 2006). In general, when measuring the spheroids diameters, there was no statistically significant change in the diameter following radiation and treatment with GNPs and DTX. However, there were trends which are useful to observe. After radiation, the GNP treated samples, whether with or without DTX, always had a slightly smaller diameter than observed in no GNPs samples. Furthermore, DTX had a large effect on the diameter of the spheroids, even in unirradiated control. Visualizing a statistically large difference in diameter is difficult, as more damage will lead to a loss in contractile forces in the cells contained within the spheroid. This leads to an initial expansion of the spheroids following damage. This is then followed by atrophy, which shrinks the tumour (Carrasco-Mantis et al. 2021). Meanwhile, healthy spheroids will maintain these contractile forces and may seem smaller despite the larger number of viable cells. The 3D viability assays done previously accounts for metabolically active cells only, and thus is more reliable of an indicator.

**Fig. 7:**
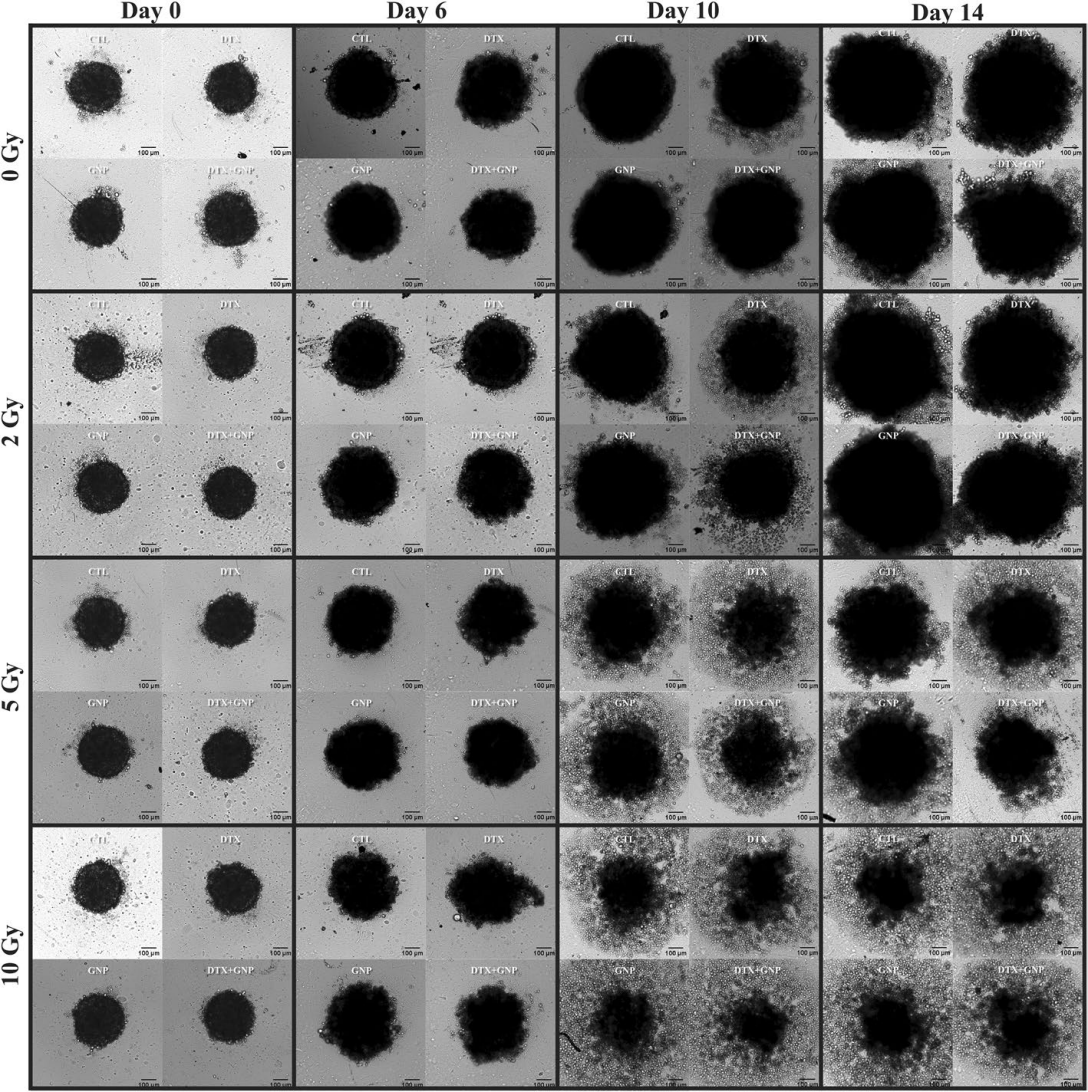
Growth of HeLa spheroids following radiation. Brightfield images of HeLa spheroids that have been untreated, treated with docetaxel, treated with gold nanoparticles, and treated with docetaxel and gold nanoparticles, and then radiated with 2 Gy, 5 Gy, and 10 Gy. Scale bar is 100 $\mathrm{\mu m}$

We can see the growth of HeLa in Fig. 7 with pictures from treatment day (day 0) to 2 weeks following treatment. As there is more damage accumulation, a lot of cell debris starts forming around the core of the spheroid. The core of the spheroid is also decimated, losing its structure, and spreading out because of loss of contractile forces in the cytoskeletons of the dead cells. For 10 Gy, the spheroids see a loss of compact structure, which leads to a larger measured diameter after day 6. The structure appears to completely fail following day 10 and day 14. These results agree with the measured proliferation results (Fig. 6a), where there are just a slim percentage of cells alive. Furthermore, we can see the effect DTX has on long term spheroid growth. The DTX-treated spheroids gather a halo of cell debris compared to control samples, which is exacerbated by radiation.

LNCaP spheroid growth can be observed in Additional file 1: Fig. S7, where we can see very tightly packed spheroids for control samples all through the 2 weeks. However, when treated with DTX, we see a loss of this tighter structure, and the growth of LNCaP is drastically delayed. As the radiation dose is increased, the spheroids do not grow very much but still maintain a structure that is indicative of a spheroid, unlike what was seen with HeLa. This also agrees to what is measured using the viability assay above (Fig. 6b). Furthermore, we see that after 14 days, the spheroids generally only have a larger amount of cell debris if treated with DTX, showing that despite the drug not appearing to largely effect the cell cycle of LNCaP, it is still able to slow growth dramatically and cause increased damage. Like HeLa, we see that the addition of GNPs did not lead to a statistically significant reduction in growth. The trend remains, however, that the samples with GNPs were smaller than their control and DTX-treated counterparts.

The data shows that DTX along with GNPs in a RT combined treatment modality can have a beneficial increase in damage to both monolayer and spheroids. However, it should be noted that the dose of DTX that was utilized has an impact on the growth of cells, in both monolayer and spheroids. When observing the overall survival, the dose of DTX can be equivalent to a dose of radiation, with the intensity depending on cell line as well as whether the platform is monolayer or spheroid. When moving to more complex in vivo models, the normal tissue toxicity will have to be managed due to this possible effect. With an irradiated combined treatment of GNPs and DTX, we can see an increase in the radiosensitivity of the cells both in spheroids and monolayer. Along with the DTX toxicity, the synergistic effect of GNPs should be carefully observed in in vivo models to ensure that the magnitude of normal tissue toxicity is within an acceptable range. However, this is far less of a worry, due to the enhanced permeability and retention (EPR) effect that allows for selective uptake of GNPs into the tumour relative to normal tissue (Kalyane et al. 2019). As a result, there will be a reduced specialized radiosensitization in the normal tissue relative to the cancer.

These results highlight the effectiveness of spheroids as a model for testing cancer treatments, including nanomaterials, such as GNPs. While the trends are similar in monolayer to spheroids, the spheroids tell a much larger and complete story. Furthermore, the spheroids have many of the tendencies that are observed in tumours, which is important when considering moving to a more personalized treatment option. We can see that LNCaP and HeLa both respond differently to both radiation as well as the addition of the combined treatment of GNPs and DTX. Using a patient’s own cells could have many benefits, by forming organoids in the lab. Organoid models have many advantages if implemented into a personalized medicine protocol. The use of a patient’s own cells allows for the maintenance of the heterogeneity present in vivo (Fatehullah et al. 2016). It should be noted that there are a few limitations with this study. First, we are using GNPs of one size and one shape. Various shapes, such as nanorods, or sizes, such as ultrasmall GNPs may prove have benefits over this studies complex. This will have to be elucidated in vivo in future mouse model studies. Second, we used common immortal cell lines to explore the effects that GNPs may have when combined with DTX and RT. This has limited practicality to the heterogenous disease that is cancer, and a wider berth of cell types and cell lines may elucidate further knowledge on the benefit of this combined treatment modality. Furthermore, by utilizing patient-specific lines in the future, the use of spheroids and organoids to screen chemoradiotherapy protocols with GNPs may enable an accurate assessment of response and allow for tailored, personalized medical care. Finally, while a small dose of DTX is utilized, there may be potential side effects when moving past the in vitro stage. Care will have to be taken to limit potential normal tissue toxicity when moving to in vivo models and eventually into clinical studies. To properly optimize GNPs with RT and DTX before moving into humans, testing in in vivo mice models both without and with immune systems to properly ascertain in a more accurate environment is part of our future experiments moving forward.

## Conclusion

By combining multiple effective treatment regimes into one, we can optimize a platform for improving patient outcome and reduce normal tissue toxicity. The use of radiosensitizers such as GNPs and cytotoxic drugs such as DTX is appealing due to their ability to synergistically work together (Bannister et al. 2020b). GNPs will increase the damage due to radiation by causing increased cascades of secondary electrons and production of ROS. Meanwhile, DTX will synchronize cells into the radiosensitive G2/M cell cycle phase, allowing the damage to more effectively build up. However, testing in a monolayer is not an effective measure of how a tumour might respond given this treatment, as it is very simplified and does not have the many complex elements present in cancer, such as a TME. Thus, using 3D structures in vitro such as a multicellular spheroid can allow for an improved platform for optimizing a treatment regime. The combined treatment of DTX and GNPs with radiation showed a statistically significant increase in damage to the monolayer as well as the spheroids. Furthermore, combining DTX with GNPs and radiation drastically changed how the spheroids grow, especially at higher doses of 5 Gy and 10 Gy. These results highlight the benefit of using spheroids for mimicking the in vivo TME. Spheroids are a far more flexible platform compared to monolayer which allow a deeper understanding of the mechanisms at hand, whether through visualization techniques or through simple viability measurements. By optimizing at a 3D level, and in the future comparing to an in vivo model, we can enable a swifter translation of GNPs to the clinic and implementation into modern RT regimes.

## Materials and methods

### Cells and culture conditions

HeLa, a cervical cancer cell line, and LNCaP, a prostate cancer cell lines, were purchased from the ATCC and have catalogue numbers CCL-2 and CRL-1740, respectively. HeLa was cultured in DMEM (Gibco) supplemented with 10% FBS (Gibco), 1% Penicillin and Streptomycin (Gibco), and 4 mM GlutaMax (Gibco). LNCaP was cultured with RPMI-1640 Medium (Gibco), supplemented with 10% Fetal Bovine Serum (Gibco), 1% Penicillin and Streptomycin (Gibco), and 2 mM GlutaMax (Gibco). For cell dissociation, TrpyLE (Gibco) was used.

### Synthesis, surface modification, and characterization of gold nanoparticles

Gold NPs of size $\approx$ 17 nm were synthesized using the citrate reduction method. This was accomplished by adding 1.18 mL of 1% ${\mathrm{HAuCl}}_{4}\cdot 3{\mathrm{H}}_{2}\mathrm{O}$ (Sigma Aldrich) to 28.82 mL of double-distilled water and heated on a hot plate while stirring vigorously. Once it reached the boiling point, 1.2 mL of 5% sodium citrate tribasic dihydrate $(\mathrm{HOC}\left(\mathrm{COONa}\right){\left({\mathrm{CH}}_{2}\mathrm{COONa}\right)}_{2}\cdot 2{\mathrm{H}}_{2}\mathrm{O}$; Sigma Aldrich) was added and mixed. Once the color of the solution changed from dark blue to red, the solution was left to boil for another 5 min while stirring. Finally, the GNP solution was brought to room temperature while stirring.

The GNPs were PEGylated using PEG of size 2000 Da, along with an RGD peptide of size 1600 Da. PEG was stirred into the GNP solutions, such that the grafting density will be 1 PEG molecule per 1 ${\mathrm{nm}}^{2}$ of surface area. For 17 nm GNPs, this results in 907 PEG per GNP to be added to the solution. Following PEGylation of GNPs, the peptide containing integrin binding domain RGD (CKKKKKKGGRGDMFG) was added to PEGylated GNPs at a ratio of 1 RGD molecule per 2 PEG molecules.

GNPs were characterized using UV–Vis spectrometry (Perkin Elmer $\uplambda$ Spectrophotometer) for approximate size and concentration estimates. Furthermore, DLS and $\upzeta$-potential (Anton Paar LiteSizer 500) were utilized to measure the hydrodynamic diameter and the surface charge of the particles. Stability of the GNPs was measured in PBS. Imaging of the GNPs with scanning transmission electron microscopy (STEM; Hitachi SU9000) was used to verify the diameter of the GNPs. Brightfield, secondary electron, and high-angle annular dark-field images of each location were taken using 14 keV STEM. Brightfield images were used for GNP diameter measurement purposes.

### Cell culture and growth of spheroids

For 2D and 3D cell models, all cells were initially split from a monolayer of cells at approximately 80% confluence. For the 2D monolayer cell model, cells were plated, such that final confluence at the end point is approximately 70% in either six-well plates or 96-well plates, with initial cell count dependent on the experiment. Once plated, cells are left in the incubator at 37 °C and 5% ${\mathrm{CO}}_{2}$ for 24 h to ensure adherence, after which experiments are initiated.

For 3D spheroid cell models, cells are plated in ultra-low attachment 96-well microplates (Corning). Diameter of spheroids were optimized before experiments by serial dilution from 50,000 cells for HeLa and 25,000 cells for LNCaP. 3,125 cells for HeLa per well and 1,000 LNCaP cells per well were found to form spheroids of approximately 300–400 $\mathrm{\mu m}$. The cells are then centrifuged at 350 $\times$ g for 5 min, at 4 °C and left in the incubator at 37 °C and 5% ${\mathrm{CO}}_{2}.$ Experiments are initiated once the spheroids form, after incubation for approximately 72 h.

### Proliferation assay of docetaxel in monolayer and spheroids

For the 2D monolayer cell model, cells were plated, such that there were 40,000 cells after 4 days, based off their doubling times. For LNCaP, this resulted in 6000 cells from LNCaP and 2500 cells from HeLa to be plated in 96 well black clear bottom microplates (Greiner), leaving one column empty for control. Once adhered, each column (8 wells) was treated with a unique docetaxel dose ranging from 250 nM to $6.4\times {10}^{-4}$ nM in media, ensuring that the concentration of dimethyl sulfoxide (DMSO) in which the DTX is stored, is constant in all columns. Similarly, for the 3D spheroid cell model, once formed to sizes of approximately 300–400 $\mathrm{\mu m}$, each column (8 wells) was treated with a unique DTX dose ranging from 250 nM down to $6.4\times {10}^{-4}$ nM. This was completed in triplicate, on three different plates per cell line. At the same time, a control column without treatment of DMSO or DTX was measured using PrestoBlue Cell Viability Reagent (Invitrogen). A 10% solution of PrestoBlue in DMEM or RPMI, depending on the cell line, was added to the cells, and left in the incubator for 1 h. The resulting fluorescence was measured using a Cytation 1 Cell Imaging Multi-Mode Reader (BioTek) for the single column.

For monolayers, the media was removed from each sample after 24 h, rinsed twice with PBS, and left to incubate for a further 48 h in fresh media. For spheroids, half of the media was removed carefully after 24 h, and then rinsed 5 times with PBS, ensuring approximately a 162 $\times$ dilution from the initial concentration. Finally, the media was replaced and left for a further 48 h in the incubator.

After 48 h, all the media for the 2D monolayer model was removed and treated with 10% PrestoBlue in media assay, following the manufacturers protocols. Plates were left in the incubator, and fluorescence was measured after 1 h using the Cytation 1. Similarly, for the 3D spheroid model, the media was removed until 100 $\mathrm{\mu L}$ remained, ensuring that the sample was not aspirated, and then 30 $\mathrm{\mu L}$ of CellTiter-Glo 3D was added to make a final concentration of 23% CellTiter-Glo 3D. The plates were then shaken for 5 min at room temperature then left for 25 min shielded from light before measuring using the Cytation 1.

### Cellular uptake of gold nanoparticle complexes

Incubation with GNP complex was done at a concentration of 10 $\upmu$g/mL for all the stated experiments in monolayer and in spheroids. To test the effect that DTX had on the uptake of the GNPs, the different cell models and cell lines were dosed with the desired concentration found in the cell proliferation experiments concurrently with GNP dosing. This correlated to a dose of 1 nM for a monolayer of LNCaP, a 4 nM for a spheroid of LNCaP, a dose of 4 nM for a monolayer of HeLa and a dose of 7 nM for a spheroid of HeLa. Samples were incubated at 37 °C and 5% ${\mathrm{CO}}_{2}$ for 24 h following treatment. Cells were then rinsed with PBS at least three times, ensuring no sample is lost while aspirating. For spheroid samples, the cells were left in Accumax (STEMCELL Technologies) for 30 min at 37 °C to improve dissociation and produce a single-cell homogenous solution, while the monolayer samples were left in TrypLE for approximately 5 min. The cells were counted using a Coulter Counter (Z2 Coulter; Beckman Coulter) for GNP quantification per cell.

To measure the gold content for each sample, 500 $\mathrm{\mu L}$ of each sample were treated with 250 $\mathrm{\mu L}$ aqua regia (3:1 ratio of $\mathrm{HCl}:{\mathrm{HNO}}_{3}$ (VWR)) in a 90 °C mineral oil bath for a minimum of 30 min. After, 100 $\mathrm{\mu L}$ of hydrogen peroxide (VWR) was added and the samples were returned to the oil bath for 30 min, ensuring complete cell breakdown. These samples were then diluted down to 2.5% v/v acid content in deionized water and the gold content was quantified using inductively coupled plasma mass spectrometry (ICP-MS; Agilent 8800 Triple Quadrupole). Spheroid samples were filtered first in a 0.2-micron filter (Fisher).

Calculation of the GNP concentration from the absolute number of Au atoms was done using the following equations:

$$\begin{aligned}\frac{\mathrm{Number\, of \,Au \,atoms}}{\mathrm{GNP}}\left[\mathrm{U}\right]&=\frac{\mathrm{Number \,of \,atoms \,per \,unit \,cell}\cdot \mathrm{Volume\, of \,GNP}}{\mathrm{Volume \,of \,unit \,cell}}\\&=4\cdot \frac{\frac{4}{3}\pi {\left(\frac{D}{2}\right)}^{3}}{{a}^{3}}\\&=\frac{2}{3}\pi {\left(\frac{D}{a}\right)}^{3}\end{aligned}$$

where *D* = core diameter of the GNP, *a* = length of a unit cell, 0.408 nm. GNPs that are synthesized using the citrate reduction method assemble into a face-centered cubic structure, which as a lattice containing 4 atoms per unit cell. This assumes a homogenous size of GNPs, which is verified through TEM imaging.

Using this, the number of GNPs per sample is calculated from

$$=\mathrm{conc}.\,\mathrm{ measured }\left[\frac{\mathrm{g}}{\mathrm{L}}\right]\cdot \mathrm{volume }\left[\mathrm{L}\right]\cdot \frac{1}{\mathrm{molar \,weight \,of\, Au}}\left[\frac{\mathrm{mol}}{\mathrm{g}}\right]\cdot {\mathrm{N}}_{\mathrm{A}}\left[\frac{\mathrm{Au}}{\mathrm{mol}}\right]\cdot \frac{1}{\mathrm{U}}\left[\frac{\mathrm{GNP}}{\mathrm{Au}}\right]$$

where ${\mathrm{N}}_{\mathrm{A}}$ is Avogadro numbers. The number of GNPs per cell is then calculated by dividing the number of GNPs by the number of cells, assuming a homogenous distribution of GNPs in the cell population.

### Preparation of cells for imaging using darkfield and hyper spectral imaging

To prepare cells for darkfield imaging for 2D monolayer samples, all cell lines were plated in a six-well plate with glass coverslips placed on the bottom of each well. The cells were then treated as described previously using GNP complex and DTX at the desired doses. Following treatment, the cells were rinsed three times with PBS and fixed using 4% PFA for 20 min at 37 °C. The cover slips were then removed from each well and mounted to a glass slide using Permount Mounting Medium (Fisher).

Similarly, for 3D spheroid samples, following treatment of spheroids with GNP complex and DTX, the cells were cleaned 5 $\times$ to dilute GNPs down dramatically, fixed in paraformaldehyde for 30 min at 37 °C, and then embedded in OCT compound (Fisher) and left in a − 20 °C freezer. The spheroids were then sectioned at 10 $\mathrm{\mu m}$ using a cryostat (Leica) and adhered to a charged microscope slide. The sections are washed with acetone to remove excess OCT, then rinsed with water. The slides are then stained with Eosin (SelecTech; Leica) stain for 30 s and washed three times with 100% ethanol. Finally, the cells were rinsed three times with xylenes, cover slipped, were mounted onto the glass slides using Permount, and dried overnight for microscopy. Each sample, either monolayer or spheroid, was imaged using darkfield microscopy and HSI (CytoViva) under a 10$\times$ and 60$\times$ objective.

### Preparation for live cell confocal imaging

For gold nanoparticle imaging, 50% of the PEG on the GNP surface was replaced with a PEG-Cy5 complex (excitation 633 nm, emission filter 650 nm LP). The GNPs were prepared otherwise the same as described previously. Glass bottom coverslip dishes (MatTek) were used to facilitate live cell imaging. Cells were first incubated with the Cy5-labelled GNPs for 24 h with the dose of DTX elucidated before. Approximately 1 h before imaging, spheroids with incubated with NucBlue Live ReadyProbe Reagent (Invitrogen) which uses Hoechst 33342 to stain the nuclei. For monolayer, this was also completed 30 min prior to imaging. Image processing was performed using ImageJ (Schindelin et al. 2012).

### Cell cycle analysis of docetaxel using flow cytometry

DTX was administered to the monolayer and spheroid cells at desired concentrations as found in proliferation assay. After set timepoints of 0, 24, 48, and 72 h, the cells were harvested as described previously using TrypLE and Accumax, and a single cell suspension was formed. Cells were washed with PBS and centrifuged at 300$\times$*g* for 5 min twice. The cell pellet was then re-suspended in 1% PFA in PBS for fixation and incubated on ice for 15 min. Cells were again washed in PBS and centrifuged at 350$\times$*g* for 5 min. Cells were re-suspended in 0.3 mL PBS and 0.7 mL freezer cold 100% ethanol (overall 70% ethanol). Samples were incubated in the dark at 4 °C for at least 1 h, further fixing and dehydrating the cell sample. For tumour samples, the tumours were treated in Collagenase/Dispase (Roche) according to manufacturer’s instructions for two hrs. Following this, the solutions were filtered through a 100-micron cell strainer and treated with the monolayer and spheroid samples.

Samples were then centrifuged at 350$\times$*g* for 10 min at 20 °C. The cell pellet was re-suspended in 1 mL of 0.5% BSA in PBS, denoted as PBS/BSA and centrifuged at 350$\times$*g* for 5 min at 20 °C. To permeabilize the cell membrane and degrade RNA, the cell pellet was re-suspended in PBTB (PBS, 0.5% BSA, 0.1% Triton-X 100) followed by an addition of RNaseA at a concentration of 100 $\upmu$ g/mL. Samples were then left to shake at 37 °C for 25 min. For labelling DNA, tubes were covered in foil, PI was added at a concentration of 10 $\upmu$g/mL and incubated on a shaker at 4 °C for at least 1 h. The cells were then centrifuged at 350$\times$*g* for 5 min at 20 °C. Finally, we re-suspended PI-stained cells in 1 mL of PBS/BSA and passed the solution through a 50 $\mathrm{\mu m}$ cell strainer before running on Flow Cytometer (BD FACS Calibur).

### Radiation of monolayer and spheroids

Cells were incubated with 10 $\upmu$g/mL of GNPs and the cell-specific doses of DTX 24 h prior to any radiation treatment. Treatment planning was done using a clinically commissioned Eclipse system (Varian Medical Systems). The plates were placed between two 30 cm $\times$ 30 cm $\times$ 5 cm thick solid water blocks with the cell layer placed at the isocenter of a 6 MV medical linear accelerator (Varian TrueBeam). A single field was used for treatment, with a beam-up gantry rotation, as the air gap between the bottom of the plate and media is much less than the air gap between the media and the top of the plate. A field size of 28 cm $\times$ 28 cm field was used, and the plane of the cells was placed directly at a source-axis distance of 100 cm, normal to the direction of the beam axis. The dose rate was set to 600 Monitor Units (MUs)/min with a reference point at isocenter at a depth of 5.0 cm. 202, 505, and 1010 MUs were delivered to the cells for doses of 2 Gy, 5 Gy, and 10 Gy, respectively. To ensure consistency, control cells were brought to the linear accelerator, set up and handled as the experimental cells, but not irradiated.

### Radiation proliferation assays

For monolayer, cells were plated, such that there are 40,000 cells after 6 days. This corresponded to 1250 cells for HeLa and 3750 cells for LNCaP. The cells were plated, such that were was at least 7 samples per condition (Control, DTX, GNP, and DTX + GNP), and measurements could be taken three separate times per plate, with each measurement having a blank well. Three 96 well plates per radiation dose were plated.

Following radiation as described above with 2 Gy, 5 Gy, and 10 Gy, samples in the first four columns were measured on the first day, then measurements were taken again on the third day and sixth day. To measure the cell proliferation, PrestoBlue Cell Viability Reagent (Invitrogen) was employed. A 10% solution of PrestoBlue in DMEM or RPMI, depending on the cell line, was added to the cells, and left in the incubator for 1 h. The resulting fluorescence was measured using a Cytation 1.

For spheroids, cells were plated, such that spheroids of 300–400 $\mathrm{\mu m}$ were formed. The cells were plated, such that were was at least 5 samples per condition (Control, DTX, GNP, and DTX + GNP), and measurements could be taken 4 separate times per plate, with each measurement having a blank well. Three 96 well plates per radiation dose were plated.

Following radiation as described above with 2 Gy, 5 Gy, and 10 Gy, samples in the first three columns were measured on the first day. Following this, measurements were taken again on the 6th day, 10th day, and 14th day. To measure the cell proliferation, CellTiter-Glo 3D (Promega) was employed. The media was removed until 100 $\mathrm{\mu L}$ remained, ensuring that the sample was not aspirated, and then 30 $\mathrm{\mu L}$ of CellTiter-Glo 3D was added to make a final concentration of 23% CellTiter-Glo 3D. The plates were then shaken for 5 min at room temperature then left for 25 min shielded from light before measuring using the Cytation 1.

For monolayers, the media was removed from each sample prior to radiation, rinsed twice with PBS, and media replaced. For spheroids, half of the media was removed carefully prior to radiation, and then rinsed 5 times with PBS, ensuring approximately a 162 $\times$ dilution from the initial concentration. Finally, the media was replaced.

### Immunofluorescence assay

Cells were grown on glass coverslips in six-well plates for 24 h prior to experimentation. Following 24 h of treatment with GNP complexes and DTX, the cells were treated with radiation as described previously at 2 Gy, 5 Gy, and 10 Gy, and incubated for 24 h at 37 °C. The cells were fixed with 4% PFA for 5 min at room temperature followed by two PBS washes for 5 min each. The cells were then blocked to reduce background noise using 2% BSA/0.1% Triton-X in PBS for 20 min. The two primary antibodies $\upgamma$ H2AX and 53BP1 were diluted 1:200 in 0.5% BSA/0.1% Triton-X/PBS, while the secondary antibody was diluted 1:500 in 0.5% BSA/0.1% Triton-X/PBS. The coverslips were placed cells-down into 50 $\mathrm{\mu L}$ of a combination of the two primary antibodies on parafilm and incubated for 1 h, followed by washing with PBS for 5 min. The cells were then rinsed twice with 0.5% BSA/0.175% Tween-20/PBS for 5 min. On a new parafilm, the cover slips were placed cells-down in 50 $\mathrm{\mu L}$ of both secondary antibodies and incubated in the dark for 30 min. Finally, the cells were rinsed in PBS, dried, mounted to glass coverslips with Prolong Glass combined with NucBlue Fixed Cell ReadyProbes nuclear stain (Invitrogen) which utilizes DAPI.

Imaging of the 53BP1 foci was performed using confocal microscopy (Zeiss LSM 980) using a 60 $\times$ oil immersion lens. The secondary antibody that attached to 53BP1 primary antibody was tagged with Alex Fluor 488 (Excitation 490 nm, Emission 525 nm), and NucBlue (Excitation 360 nm, Emission 460 nm) stained the nucleus. All conditions including acquisition settings used between experiments was maintained constant.

### Growth analysis of radiated spheroids

Spheroids were plated for radiation as described above. Brightfield images of the spheroids were taken every 2 days following radiation using the Cytation 1 using a 4 $\times$ objective. To calculate the average diameter of a spheroid, ImageJ was used. An outline of the spheroid was drawn and then the area was calculated. From this area, the diameter was calculated using $d=\sqrt{\frac{A}{\pi }}$. Outlines of the spheroids were drawn manually and did not include cell debris that appeared due to damage from either DTX or radiation. A minimum of 5 and a maximum of 20 spheroids were used per timepoint to calculate the average diameter of each condition.

### Hypoxic analysis of spheroids

A wide range of spheroid sizes were grown. For HeLa, 200 $\mathrm{\mu m}$ to 1000 $\mathrm{\mu m}$ were grown and for LNCaP, 280 $\mathrm{\mu m}$ to 1100 $\mathrm{\mu m}$ spheroids were grown. The spheroids were then incubated with 10 $\mathrm{\mu M}$ of Image-iT Hypoxia Reagent (Invitrogen) and then were incubated for 1 h at 37 °C. The media was then exchanged with fresh medium, DMEM or RPMI depending on cell line. Brightfield images of the spheroids were then taken using the Cytation 1 using a 4$\times$ objective. Furthermore, a GFP filter set (Excitation 469/35 nm, Emission 525/39 nm) was used to image the hypoxia reagent (Excitation 488 nm, Emission 520 nm).

### Statistical analysis

A Welch's independent *t* test with Bonferroni correction was preformed using the statannot python package. A *p* value < 0.05 was considered statistically significant. Experiments were repeated three times and the data presented is the average, for all experiments.

### Supplementary Information

**Additional file 1.** **Figure S1**. Characterizing spheroid size and gold nanoparticle stability. (a) Size of the spheroids for HeLa and LNCaP under different initial cell count conditions. An approximate size of 300-400 μm was used for all experiments. (b) Brightfield images of the spheroids under different initial cell counts, scale bar is 250 μm. (c) Gold nanoparticles conjugated with polyethylene glycol and a peptide containing integrin binding domain RGD is stable after 2 months while stored at 4 °C. **Figure S2.** Docetaxel characterization in LNCaP. (a,b) Proliferation assays for a (a) two-dimensional monolayer and a (b) three-dimensional spheroid, for the prostate cancer cell line LNCaP, treated with docetaxel. (c,d) Cell cycle analysis of a (c) monolayer and (d) spheroids of LNCaP cells treated with the GR50 dose of docetaxel, calculated per modality. **Figure S3.** Darkfield images of monolayer and spheroids. (a,b) Darkfield images of a monolayer of HeLa (a) without and (b) with docetaxel. Cells with multinucleated cells due to docetaxel have been circled in red on (b). Scale bar is 40 μm. (c,d) Darkfield images of 10 μm sections of HeLa spheroids (c) without and (d) with docetaxel. Inset is hyper spectral spectrum of cells. Scale bar is 40 μm. **Figure S4.** Confocal images of 53BP1 foci in monolayer of HeLa. Images of HeLa nuclei 24 hours after being irradiated with 2 Gy, 5 Gy, and 10 Gy. Samples include untreated, docetaxel treated, gold nanoparticle treated, and combined docetaxel and gold nanoparticle treatment. Nuclei and 53BP1 foci are marked in blue and green, respectively. Scale bar is 25 μm. **Figure S5.** Confocal images of 53BP1 foci in monolayer of LNCaP. Images of LNCaP nuclei 24 hours after being irradiated with 2 Gy, 5 Gy, and 10 Gy. Samples include untreated, docetaxel treated, gold nanoparticle treated, and combined docetaxel and gold nanoparticle treatment. Nuclei and 53BP1 foci are marked in blue and green, respectively. Scale bar is 25 μm. **Figure S6.** Hypoxia analysis in spheroids. (a,b) Analysis of hypoxic core in spheroids of (a) experimental size (diameter ≈300–400 μm) and (b) spheroids of diameter > 600 μm. Due to radiations ability to create reactive oxygen species, a core that is normoxic is desirable to do more damage to all the cells. Scale bar is 300 μm. **Figure S7.** Growth of LNCaP spheroids following radiation. Brightfield images of LNCaP spheroids that have been untreated, treated with docetaxel, treated with gold nanoparticles, and treated with docetaxel and gold nanoparticles, and then radiated with 2 Gy, 5 Gy, and 10 Gy. Scale bar is 100 μm.

## Data Availability

The data sets used and/or analysed during the current study are available from the corresponding author on reasonable request.

## Abbreviations

2D: Two dimensional
3D: Three dimensional
GNPs: Gold nanoparticles
DTX: Docetaxel
RT: Radiotherapy
DSBs: Double strand breaks
TME: Tumour Microenvironment
ECM: Extracellular matrix
IMRT: Intensity modulated radiotherapy
IGRT: Image guided radiotherapy
PEG: Polyethylene glycol
HSI: Hyperspectral Imaging
DLS: Dynamic light scattering
MV: Megavoltage
NHEJ: Non-homologous end joining
ROS: Reactive oxygen species
ICP-MS: Inductively coupled plasma mass spectrometry
