# Supplementary material for: Utilizing two-dimensional monolayer and three-dimensional spheroids to enhance radiotherapeutic potential by combining gold nanoparticles and docetaxel
Source: Cancer Nanotechnol. 2023 Oct 19;14(1):80. doi: 10.1186/s12645-023-00231-5 (PMC10587262; doi:10.1186/s12645-023-00231-5)
Supplement: Supplementary file 1 — Additional file 1. Figure S1. Characterizing spheroid size and gold nanoparticle stability. (a) Size of the spheroids for HeLa and LNCaP under different initial cell count conditions. An approximate size of 300-400 μm was used for all experiments. (b) Brightfield images of the spheroids under different initial cell counts, scale bar is 250 μm. (c) Gold nanoparticles conjugated with polyethylene glycol and a peptide containing integrin binding domain RGD is stable after 2 months while stored at 4 °C. Figure S2. Docetaxel characterization in LNCaP. (a,b) Proliferation assays for a (a) two-dimensional monolayer and a (b) three-dimensional spheroid, for the prostate cancer cell line LNCaP, treated with docetaxel. (c,d) Cell cycle analysis of a (c) monolayer and (d) spheroids of LNCaP cells treated with the GR50 dose of docetaxel, calculated per modality. Figure S3. Darkfield images of monolayer and spheroids. (a,b) Darkfield images of a monolayer of HeLa (a) without and (b) with docetaxel. Cells with multinucleated cells due to docetaxel have been circled in red on (b). Scale bar is 40 μm. (c,d) Darkfield images of 10 μm sections of HeLa spheroids (c) without and (d) with docetaxel. Inset is hyper spectral spectrum of cells. Scale bar is 40 μm. Figure S4. Confocal images of 53BP1 foci in monolayer of HeLa. Images of HeLa nuclei 24 hours after being irradiated with 2 Gy, 5 Gy, and 10 Gy. Samples include untreated, docetaxel treated, gold nanoparticle treated, and combined docetaxel and gold nanoparticle treatment. Nuclei and 53BP1 foci are marked in blue and green, respectively. Scale bar is 25 μm. Figure S5. Confocal images of 53BP1 foci in monolayer of LNCaP. Images of LNCaP nuclei 24 hours after being irradiated with 2 Gy, 5 Gy, and 10 Gy. Samples include untreated, docetaxel treated, gold nanoparticle treated, and combined docetaxel and gold nanoparticle treatment. Nuclei and 53BP1 foci are marked in blue and green, respectively. Scale bar is 25 μm. Figure S6. Hypoxi [file 12645_2023_231_MOESM1_ESM.docx]

**Utilizing two-dimensional monolayer and three-dimensional spheroids to enhance radiotherapeutic potential by combining gold nanoparticles and docetaxel**

**Kyle Bromma^1^, *PhD*, Wayne Beckham^1,2^, *PhD*, Devika B. Chithrani^1,2,3,4,*^, *PhD***

1. **Department of Physics and Astronomy, University of Victoria, Victoria, BC, Canada.**
2. **British Columbia Cancer, Victoria, BC, Canada.**
3. **Centre for Advanced Materials and Related Technologies (CAMTEC), University of Victoria, Victoria, BC, Canada.**
4. **Division of Medical Sciences, University of Victoria, Victoria, BC, Canada**

*** Corresponding author. Email: devikac@uvic.ca**

**Additional file**


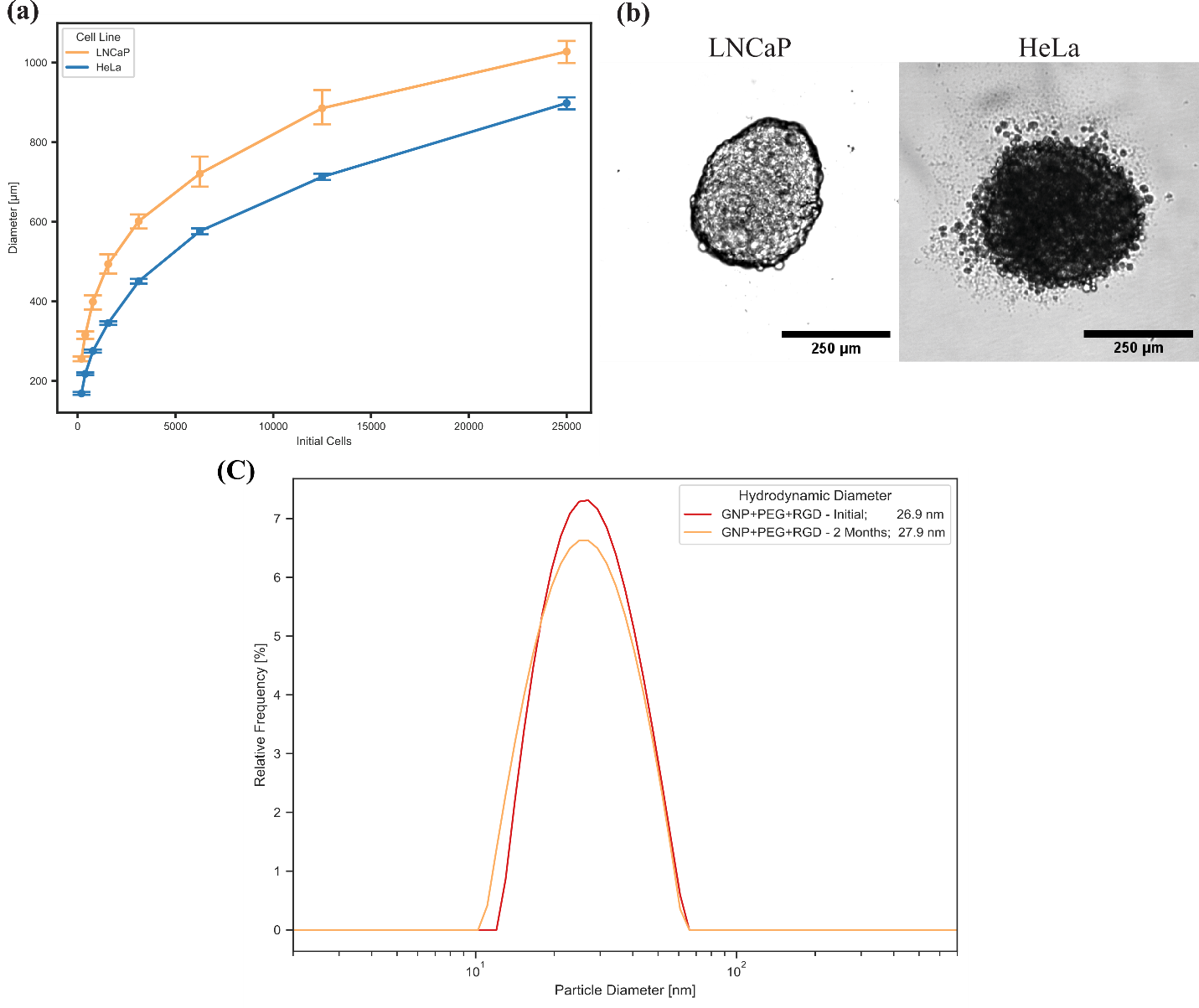


**Figure S1. Characterizing spheroid size and gold nanoparticle stability.** (a) Size of the spheroids for HeLa and LNCaP under different initial cell count conditions. An approximate size of 300-400 $\mu$m was used for all experiments. (b) Brightfield images of the spheroids under different initial cell counts, scale bar is 250 $\mu$m. (c) Gold nanoparticles conjugated with polyethylene glycol and a peptide containing integrin binding domain RGD is stable after 2 months while stored at 4 ^o^C.


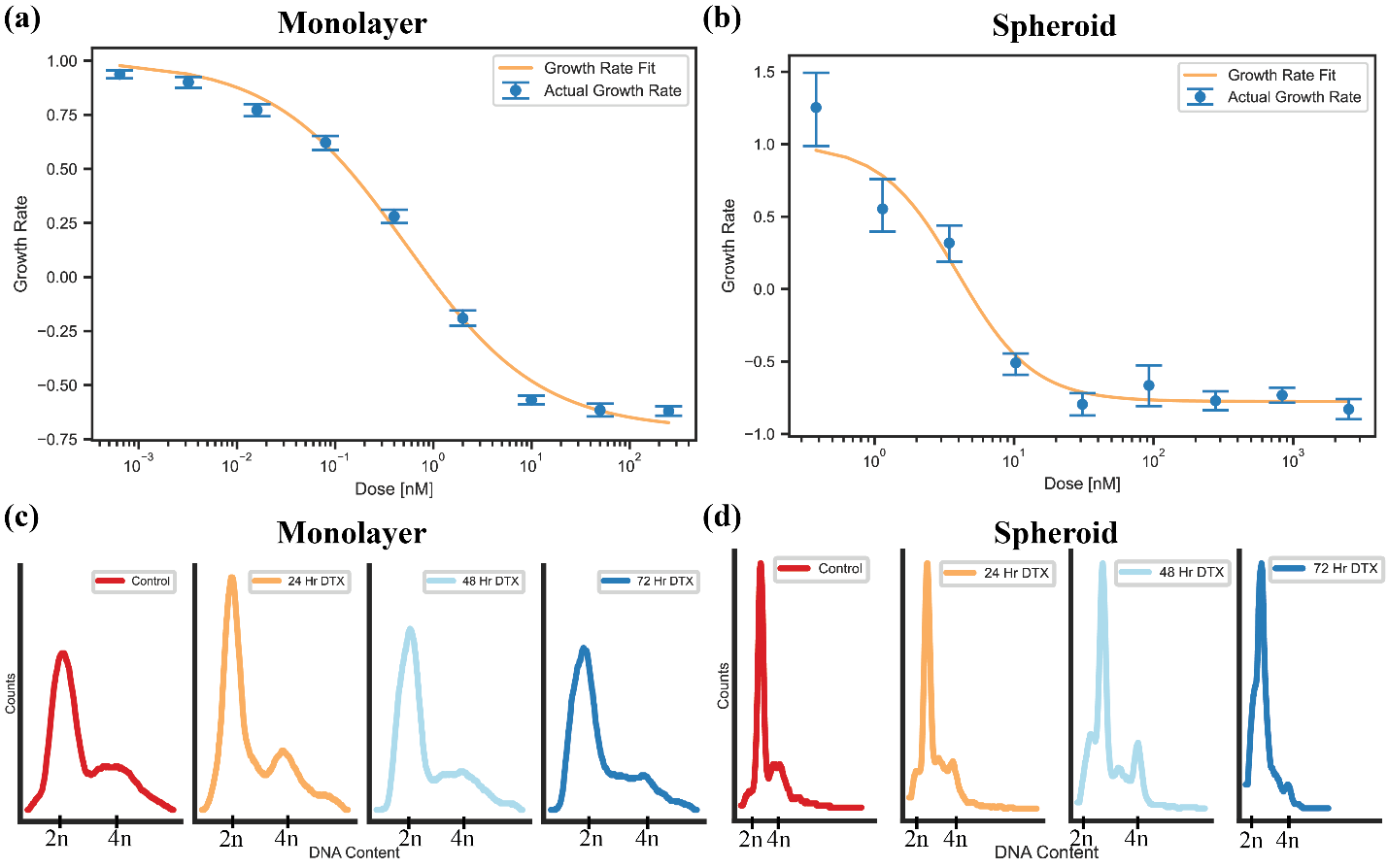


**FigureS 2. Docetaxel characterization in LNCaP.** (a,b) Proliferation assays for a (a) two-dimensional monolayer and a (b) three-dimensional spheroid, for the prostate cancer cell line LNCaP, treated with docetaxel. (c,d) Cell cycle analysis of a (c) monolayer and (d) spheroids of LNCaP cells treated with the GR50 dose of docetaxel, calculated per modality.


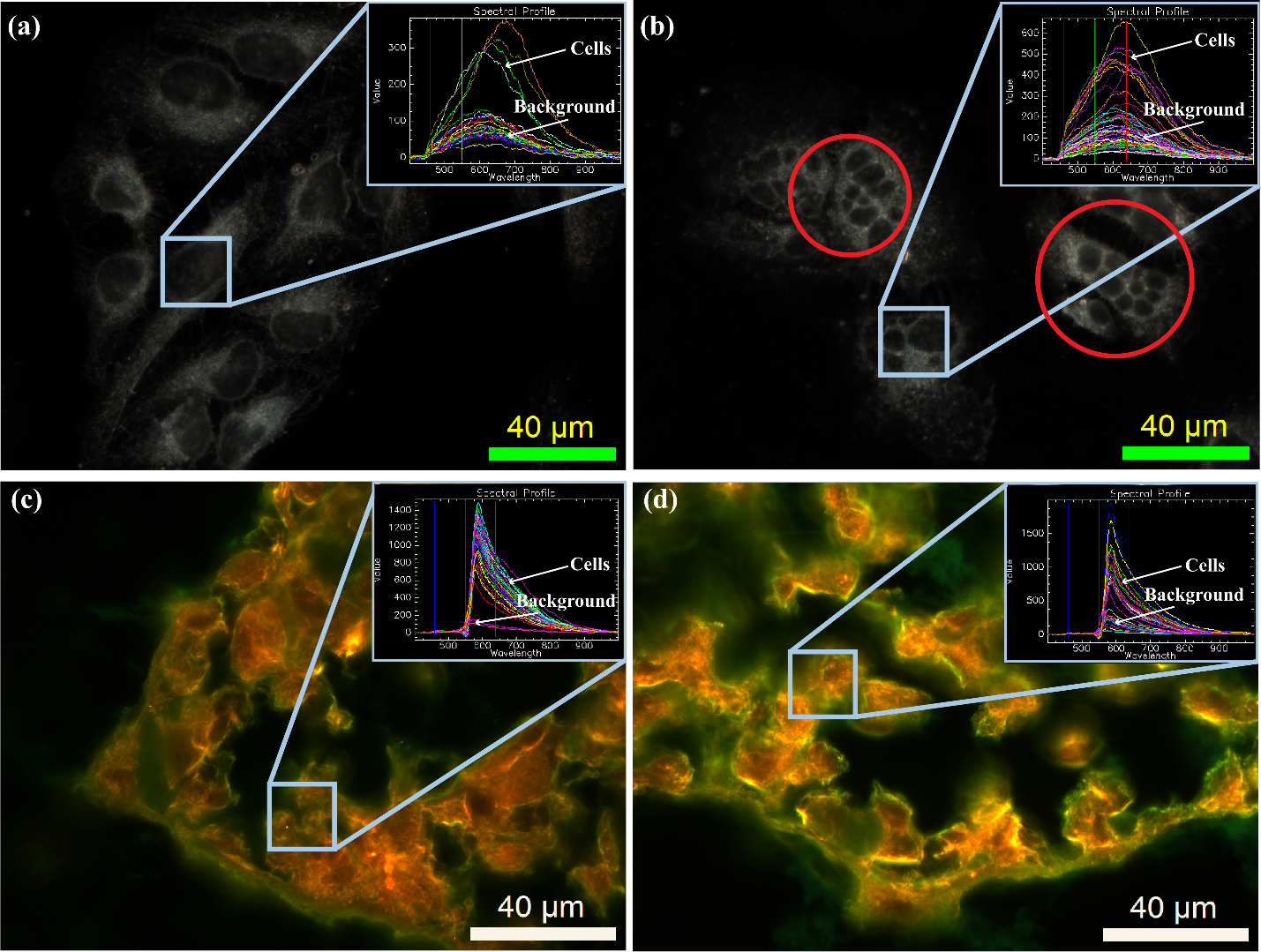


**Figure S3. Darkfield images of monolayer and spheroids.** (a,b) Darkfield images of a monolayer of HeLa (a) without and (b) with docetaxel. Cells with multinucleated cells due to docetaxel have been circled in red on (b). Scale bar is 40 $\mu$m. (c,d) Darkfield images of 10 $\mu$m sections of HeLa spheroids (c) without and (d) with docetaxel. Inset is hyper spectral spectrum of cells. Scale bar is 40 $\mu$m.


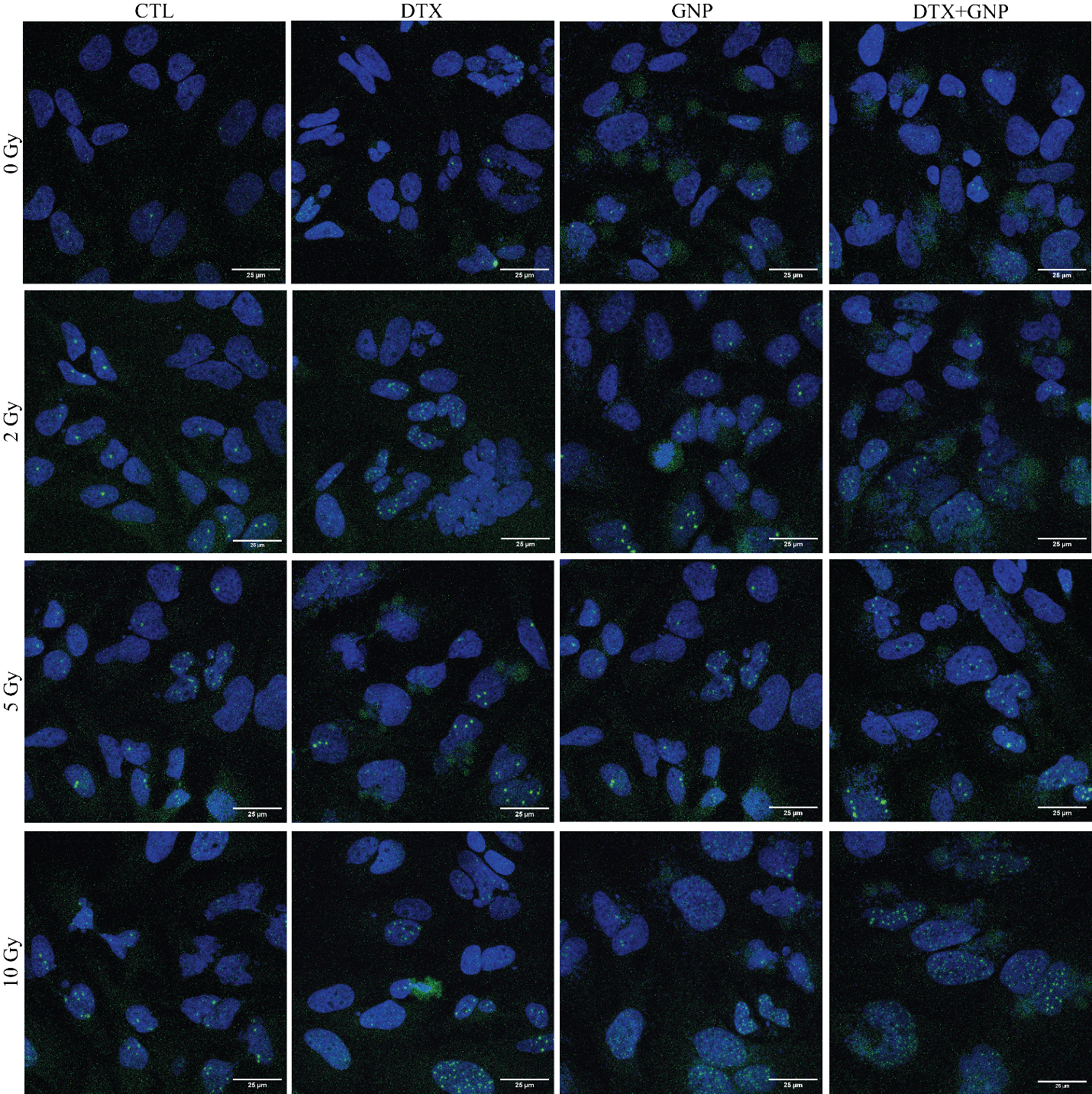


**Figure S4. Confocal images of 53BP1 foci in monolayer of HeLa.** Images of HeLa nuclei 24 hours after being irradiated with 2 Gy, 5 Gy, and 10 Gy. Samples include untreated, docetaxel treated, gold nanoparticle treated, and combined docetaxel and gold nanoparticle treatment. Nuclei and 53BP1 foci are marked in blue and green, respectively. Scale bar is 25 $\mu$m.


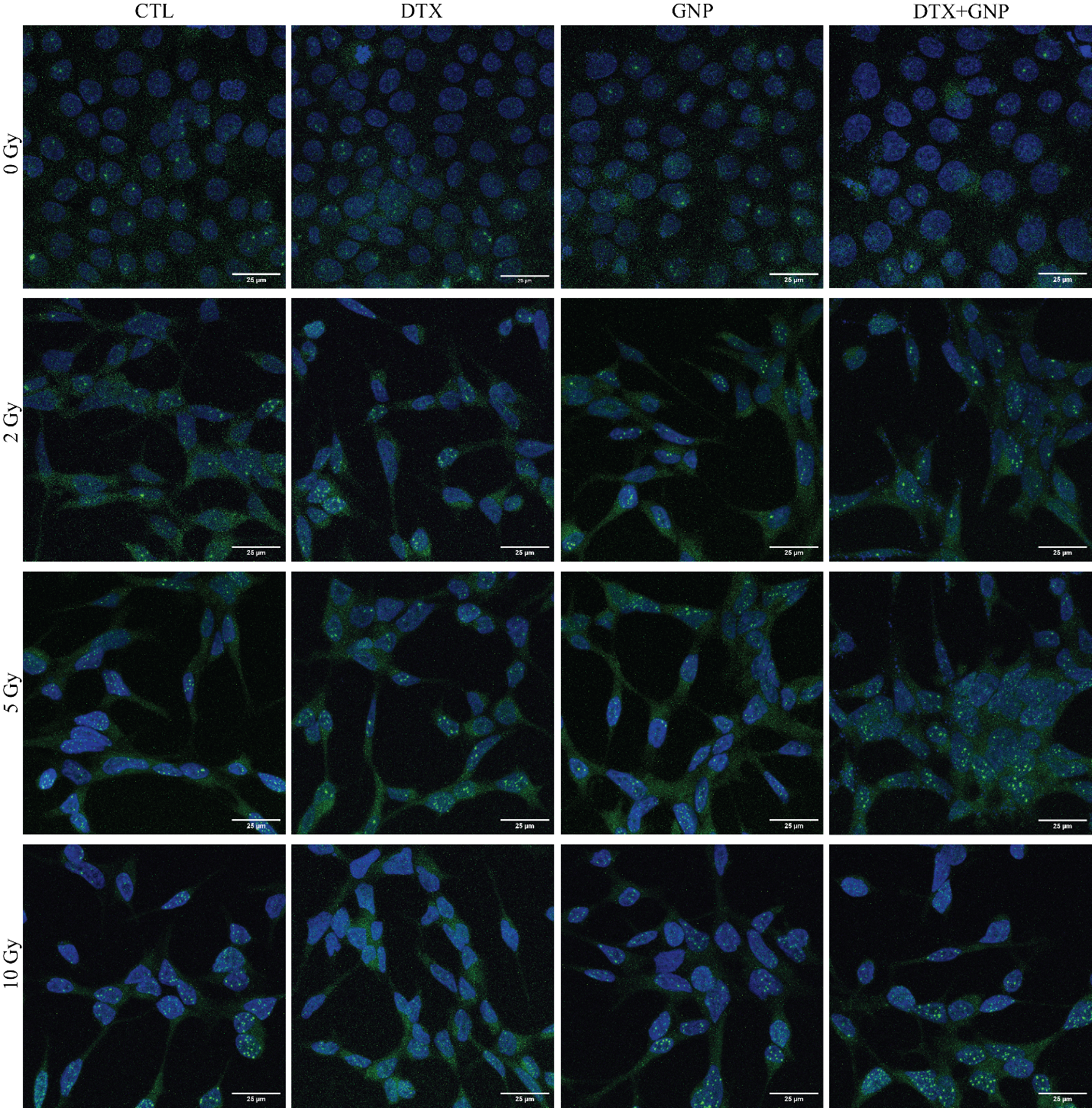


**Figure S5. Confocal images of 53BP1 foci in monolayer of LNCaP.** Images of LNCaP nuclei 24 hours after being irradiated with 2 Gy, 5 Gy, and 10 Gy. Samples include untreated, docetaxel treated, gold nanoparticle treated, and combined docetaxel and gold nanoparticle treatment. Nuclei and 53BP1 foci are marked in blue and green, respectively. Scale bar is 25 $\mu$m.


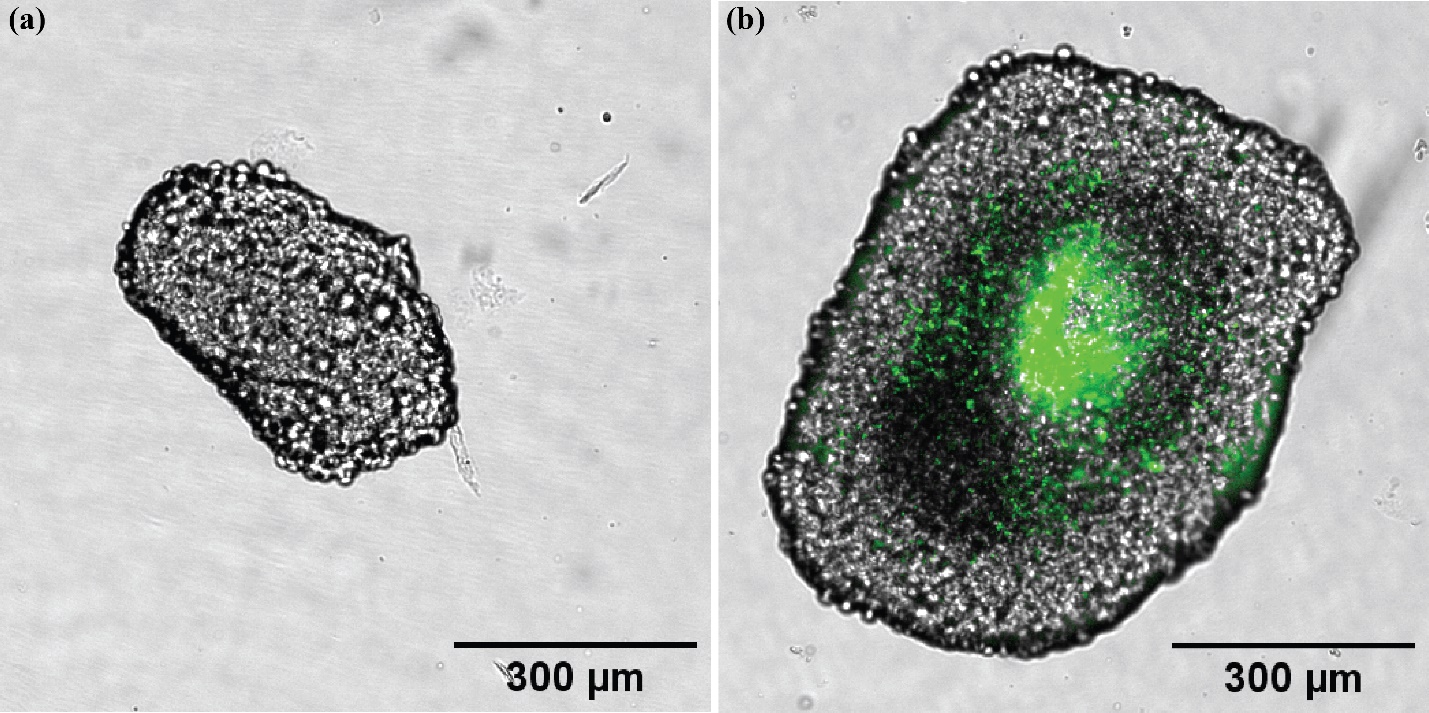


**Figure S6. Hypoxia analysis in spheroids.** (a,b) Analysis of hypoxic core in spheroids of (a) experimental size (diameter $\approx$300-400 $\mu$m) and (b) spheroids of diameter >600$\mu$m. Due to radiations ability to create reactive oxygen species, a core that is normoxic is desirable to do more damage to all the cells. Scale bar is 300 $\mu$m.

*
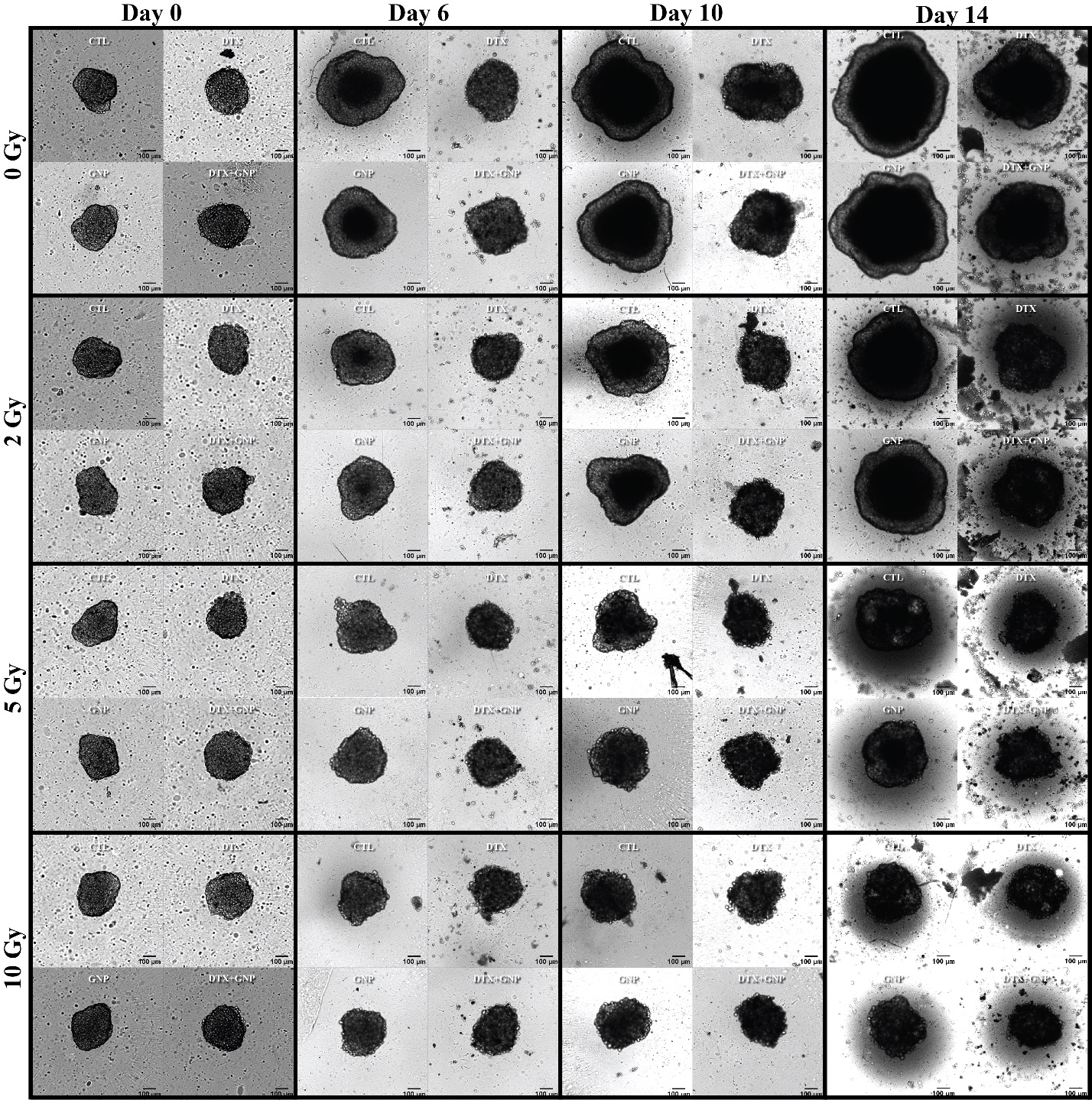
*

**Figure S7. Growth of LNCaP spheroids following radiation.** Brightfield images of LNCaP spheroids that have been untreated, treated with docetaxel, treated with gold nanoparticles, and treated with docetaxel and gold nanoparticles, and then radiated with 2 Gy, 5 Gy, and 10 Gy. Scale bar is 100 $\mu$m.
